# Supplementary material for: Use of Omics Data in Fracture Prediction; a Scoping and Systematic Review in Horses and Humans
Source: Animals (Basel). 2021 Mar 30;11(4):959. doi: 10.3390/ani11040959 (PMC8065418; doi:10.3390/ani11040959)
Supplement: Supplementary file 1 [file animals-11-00959-s001.pdf]

animals

MDPI

Supplementary Materials: Use of Omics Data in Fracture Prediction; a Scoping and Systematic Review in Horses and Humans

Seungmee, Lee, Melissa Baker, Michael Clinton and Sarah E.Taylor

Table S1. Inclusion and exclusion criteria for the three primary outcomes.

Section and topic	Item No	Checklist item	Pages
**ADMINISTRATIVE INFORMATION**			
Title:			
Identification	1a	Identify the report as a protocol of a systematic review	6
Update	1b	If the protocol is for an update of a previous systematic review, identify as such	6
Registration	2	If registered, provide the name of the registry (such as PROSPERO) and registration number	NA
Authors:			
Contact	3a	Provide name, institutional affiliation, e-mail address of all protocol authors; provide physical mailing address of corresponding author	1
Contributions	3b	Describe contributions of protocol authors and identify the guarantor of the review	28
Amendments	4	If the protocol represents an amendment of a previously completed or published protocol, identify as such and list changes; otherwise, state plan for documenting important protocol amendments	7-9
Support:			
Sources	5a	Indicate sources of financial or other support for the review	28
Sponsor	5b	Provide name for the review funder and/or sponsor	28
Role of sponsor or funder	5c	Describe roles of funder(s), sponsor(s), and/or institution(s), if any, in developing the protocol	28
**INTRODUCTION**			
Rationale	6	Describe the rationale for the review in the context of what is already known	5,6
Objectives	7	Provide an explicit statement of the question(s) the review will address with reference to participants, interventions, comparators, and outcomes (PICO)	6
**METHODS**			
Eligibility criteria	8	Specify the study characteristics (such as PICO, study design, setting, time frame) and report characteristics (such as years considered, language, publication status) to be used as criteria for eligibility for the review	7, Table S1-S5
Information sources	9	Describe all intended information sources (such as electronic databases, contact with study authors, trial registers or other grey literature sources) with planned dates of coverage	7
Search strategy	10	Present draft of search strategy to be used for at least one electronic database, including planned limits, such that it could be repeated	7
Study records:			
Data management	11a	Describe the mechanism(s) that will be used to manage records and data throughout the review	8/9, Fig 1

Selection process	11b	State the process that will be used for selecting studies (such as two independent reviewers) through each phase of the review (that is, screening, eligibility and inclusion in meta-analysis)	8/9, Fig 1
Data collection process	11c	Describe planned method of extracting data from reports (such as piloting forms, done independently, in duplicate), any processes for obtaining and confirming data from investigators	9
Data items	12	List and define all variables for which data will be sought (such as PICO items, funding sources), any pre-planned data assumptions and simplifications	Table S14
Outcomes and prioritization	13	List and define all outcomes for which data will be sought, including prioritization of main and additional outcomes, with rationale	Table S12-S13
Risk of bias in individual studies	14	Describe anticipated methods for assessing risk of bias of individual studies, including whether this will be done at the outcome or study level, or both; state how this information will be used in data synthesis	9, Table-S12
Data synthesis	15a	Describe criteria under which study data will be quantitatively synthesised	NA
	15b	If data are appropriate for quantitative synthesis, describe planned summary measures, methods of handling data and methods of combining data from studies, including any planned exploration of consistency (such as I^2, Kendall's τ)	NA
	15c	Describe any proposed additional analyses (such as sensitivity or subgroup analyses, meta-regression)	NA
	15d	If quantitative synthesis is not appropriate, describe the type of summary planned	21
Meta-bias(es)	16	Specify any planned assessment of meta-bias(es) (such as publication bias across studies, selective reporting within studies)	NA
Confidence in cumulative evidence	17	Describe how the strength of the body of evidence will be assessed (such as GRADE)	11

*PRISMA-P (Preferred Reporting Items for Systematic review and Meta-Analysis Protocols) 2015 checklist: recommended items to address in a systematic review protocol.; It is strongly recommended that this checklist be read in conjunction with the PRISMA-P Explanation and Elaboration (cite when available) for important clarification on the items. Amendments to a review protocol should be tracked and dated. The copyright for PRISMA-P (including checklist) is held by the PRISMA-P Group and is distributed under a Creative Commons Attribution Licence 4.0.[30]

Table S2. Inclusion and exclusion criteria for primary outcome—changes in microRNAs/ peptide analysis/ gene expression related to musculoskeletal injuries in horses.

Criteria	Inclusion	Exclusion
Population	All breeds and ages of domesticated horses	Other types of domesticated equids (donkeys and mules)
Interventions	Musculoskeletal injuries E.g. laminitis, osteochondrosis, tendon injury; RCT treated with miRNA-29 or a placebo	Previous laminitis or tendon injury were excluded based in history and clinical examinations
Comparator	Healthy control without any musculoskeletal injuries	Any horses with a septic condition
Outcome	Differentially expressed miRNA/mRNA, protein expression Assessment of accuracy of diagnostic ability	No differentiation between musculoskeletal disease and control groups
Language	Publications in the English language	Publications not in the English language
Study design	Cohort, case-control, randomized controlled trials, cross-sectional studies	Case series, case reports, narrative reviews, textbook chapters
Publication type	Peer-reviewed publications	Unable to obtain full study details

Table S3. Inclusion and exclusion criteria for primary outcome—microRNAs and their targets in response to exercise and mechanical loading in horses and humans.

Criteria	Inclusion	Exclusion
Population	All breeds of domesticated horses	Other types of domesticated equids (donkeys and mules)
Interventions	Exercise and / mechanical loading<br>Human study sprint training	History or current clinical signs of cardiovascular, pulmonary, metabolic or bone diseases; traumatic fractures in the 2 previous years; routine involvement in intermittent sports
Comparator	No exercise or mechanical loading<br>Human study control group not subjected to sprint training	
Outcome	Differentially expressed miRNA/mRNA, protein expression<br>Assessment of accuracy of diagnostic ability	No differentiation between exercise/mechanical loading and control groups
Language	Publications in the English language	Publications not in the English language
Study design	Cohort, case-control, randomized controlled trials, cross-sectional studies	Case series, case reports, narrative reviews, textbook chapters
Publication type	Peer-reviewed publications	Unable to obtain full study details

Table S4. Inclusion and exclusion criteria for primary outcome—genetic association studies related to stress fractures in horses or young adults (e.g. athletes/military recruits) equivalent to equine stress fractures.

Criteria	Inclusion	Exclusion
Population	Thoroughbred horses in active race training either flat or national hunt (one horse study)<br>Military recruit or elite athletes (professional or competing at international level, aged 18-35yrs.	Other breeds of horse or domesticated equids (donkeys and mules).<br>Non-military recruit or non-elite athlete.
Interventions	Horse study fracture of the distal limb<br>Human studies stress fractures diagnosed with radiography, scintigraphy, CT or MRI	Horse study; no fracture or fractures of the proximal limb or axial skeleton.<br>No imaging diagnosis of stress fracture, concurrent disease such as anorexia
Comparator	Horses with no stress fracture over 4 years of age in active race training	Horses less than 4 years or not in race training.
Outcome	Single nucleotide polymorphisms	No differentiation between stress fracture and control groups
Language	Publications in the English language	Publications not in the English language
Study design	Cohort, case-control, randomized controlled trials, cross-sectional studies	Case series, case reports, narrative reviews, textbook chapters
Publication type	Peer-reviewed publications	Unable to obtain full study details

Table S5. Inclusion and exclusion criteria for secondary outcome—miRNA and their target analysis related to osteoporotic fragility fracture in humans.

Criteria	Inclusion	Exclusion
Population	Premenopausal women (<50 years) with idiopathic osteoporosis, postmenopausal women (>50 years) with osteoporosis, men (30–55years) with osteoporosis, controls were men and women (30–72 years) that sustained fragility fractures with normal bone mineral density or osteopenia	Osteoporosis secondary to other conditions such as diabetes mellitus, diseases arthritis, psoriatic arthritis, systemic lupus erythematosus, Crohn's disease, ulcerative colitis), COPD, chronic kidney and liver
Interventions	Incomplete stress fracture or complete fracture of any skeleton	No mention of fracture
Comparator	Osteoporotic but no known fracture	
Outcome	Differentially expressed miRNA/mRNA, protein expression Assessment of accuracy of diagnostic ability	Non exclusion of haemolytic blood samples
Language	Publications in the English language	Publications not in the English language
Study design	Cohort, case-control, randomized controlled trials, cross-sectional studies	Case series, case reports, narrative reviews, textbook chapters
Publication type	Peer-reviewed publications	Unable to obtain full study details

Table S6. Detailed data extraction spreadsheet of selected original research paper—Study design and population.

Author	Title	Subcatego-rised (Species)	Study design	Study population Case	Sample size Case	Age Case	Study population Controls	Sample size Controls	Age Controls	Sample size consideratio n Y/N
Arai *et al.* (2008)	Analysis of Cartilage oligomeric matrix protein (COMP) in synovial fluid, serum and urine from 51 racehorses with carpal bone fracture	1a (Horse)	CS	Thoroughbreds with osteochondral fractures of the carpal joints with osteophyte positive	26	NA	osteochondral fractures of the carpal joints with osteophyte negative	25	NA	N
Arens *et al.* (2013)	Comparison of the use of scapular ultrasonography, physical examination, and measurement of serum biomarkers of bone turnover versus scintigraphy for detection of bone fragility syndrome in horses	1a (Horse)	CC	20 horses with bone fragility disorder (BFS) or silicate-associated OP	20	14 years (range, 5 to 25 years	control horses (various breeds)	28	14 years (range, 5 to 25 years	N
Blott *et al.* (2014)	A genome-wide association study demonstrates significant genetic variation for fracture risk in	1c (Horses)	CC	Thoroughbred horse with catastrophic distal limb fracture	269	NA	Controls horses	253	NA (> 4 years)	Y

	Thoroughbred racehorses									
Cappelli *et al.* (2018)	Circulating miRNAs as putative biomarkers of exercise adaptation in endurance horses	1b (Horse)	QE	Arabian horses 2 h after the end of competition T1	4	6.7 (6–9)	Arabian horses Before competition T0	6.7 (6–9)	4	N
Chatzipapas *et al.* (2009)	Polymorphisms of the Vitamin D Receptor Gene and Stress Fractures	1c (Human)	CC	male military personnel with SF	32	22.91 ± 2.99	healthy volunteers	32	22.91 ± 3.21	N
Cleary *et al.* (2010)	Effect of exercise and osteochondral injury on synovial fluid and serum concentrations of carboxy-terminal telopeptide fragments of type II collagen in racehorses	1a (Horse)	CC	Thoroughbreds with osteochondral injury	38	Case (2 to 7)	Thoroughbreds with osteochondral injury<br>1)pre-exercise<br>2)post-exercise	40	Control (14 to 21 months)	N
Desjardin *et al.* (2014)	Next-generation sequencing identifies equine cartilage and subchondral bone miRNAs and suggests their involvement in osteochondrosis physiopathology	1a (Horse)	QE	Anglo-Arabian foals Osteochondrosis (OC)	Animal N = 3 from total 37 horses, Tissues N = 15	10-month	Anglo-Arabian foals healthy	Animal N =3 from total 37 horses, Tissues N = 15	10-month	N
Farries *et al.* (2019)	Expression quantitative trait loci in equine skeletal	1b (Horse)	QE	Healthy After training	111	757.5 days	Healthy before training	111	611.7 days	N

	muscle reveals heritable variation in metabolism and the training responsive transcriptome									
Feichtinger *et al.* (2018)	Bone-related circulating microRNAs miR-29b-3p, miR-550a-3p, and miR-324-3p and their association to bone microstructure and histomorphometry	2 (Human)	CS	Premenopausal (Pre-MP) / male idiopathic with low traumatic fractures Caucasian	Pre-MP N = 10<br>Male N = 16	39.0 ± 8.6<br>43.7 ± 11.1	Postmenopausal (P-MP) osteoporosis (OP)	P-MP N =10	59.0 ± 11.4	N
Feurer *et al.*(2019)	Lack of association between select circulating miRNAs and bone mass, turnover, and fractures: data from the OFELY cohort	2 (Human)	CS	P-MP F (122 women with fracture)	583	68.2	Pre-MP F (1 woman with fracture)	99	48.7	N
Friedman *et al.* (2014)	Novel candidate genes putatively involved in stress fracture predisposition detected by whole-exome sequencing	1c (Human)	CC	soldiers with high grade SF	N = 34<br>For validation 104 SF cases	20.1 ± 0.9	age-matched soldiers with no evidence of SF	N = 60<br>For validation 72 controls	20.1±1.1	N
Frisbie *et al.* (2010)	Serum biomarker levels for musculoskeletal disease in	1a (Horse)	C	Musculoskeletal injury including intra-articular fragmentation (IAF), tendon or	59	2–3	Without injury	71	2-3	N

	two- and three-year-old racing Thoroughbred horses: A prospective study of 130 horses			ligamentous injury (TL), stress fractures (SF) and dorsal metacarpal disease (DMD). Monthly longitudinal samples						
Hakansson *et al.* (2018)	Circulating small non-coding RNAs as biomarkers for recovery after exhaustive or repetitive exercise	1b (Human)	RCT	Elite endurance cyclists After exhaustive endurance exercise (EEE) Peripheral Artery Disease (PAD) for impaired muscle recovery Time Trial (TT60) Cohort	8<br>16<br>13	22.9 ± 1.2<br>69 ± 13<br>27.7 ± 1.2	Eight male elite endurance cyclists Before EEE	8	22.9 ± 1.2	N
Horak *et al.* (2018)	Exercise-induced circulating microRNA changes in athletes in various training scenarios	1b (Human)	QE	young male athletes explosive 3 exercises week 5 and week 8	30 week 5 and week 8	22.5 ± 4.06	week 0	30	22.5 ± 4.06	N
Jackson *et al.* (2009)	Bone biomarkers and risk of fracture in two- and three-year old Thoroughbreds	1a (Horse)	C	2- and 3-year-olds injury in the subsequent flat racing season	Two-year-old study 60 Three-year-old study 30	2<br>3	No-injury	469<br>296	2<br>3	N

Jackson *et al* (2015)	Relationship between serum biomarkers of cartilage and bone metabolism and joint injury in young Thoroughbred racehorses in training	1a (Horse)	C	Thoroughbred racehorses with a carpal or fetlock joint injury	50	2–3-year-old	uninjured horses (control horses)	50	2–3-year-old	Y
Kim *et al.* (2018)	Integrated analysis of microRNA and mRNA expressions in peripheral blood leukocytes of Warmblood horses before and after exercise	1b (Horse)	QE	Warmblood after exercise	N = 4	11 (7–15)	before exercise	N = 4	11 (7–15)	N
Kocijan *et al.* (2016)	Circulating microRNA signatures in patients with idiopathic and postmenopausal osteoporosis and fragility fractures	2 (Human)	CS	Patients With Idiopathic OP (Pre-MP, man, P-MP)	36	46.6 ± 13.0	Healthy control	39	46.6 ± 9.4	N
Korvala *et al.* (2010)	Genetic predisposition for femoral neck stress fractures in military conscripts	2 (Human)	CC	military conscripts with a femoral neck SF	72	20.3 ± 1.6	military conscripts without SF	120	18.9 ± 0.5	N
Kuemmerle *et al*. (2016)	Identification of novel Equine (Equus caballus) tendon markers using RNA sequencing	1a (Horse)	CS	tendon from the forelimb region of the SDFT post-mortem	6 Warmblood (5) Lipizzaner (1)	7.3 ± 4.6	cancellous bone, cartilage, cervical dermal tissue.	6 Warmblood (5) Lipizzaner (1)	7.3 ± 4.6	N

Ladang *et al* (2019)	Evaluation of a Panel of MicroRNAs that Predicts Fragility Fracture Risk: A Pilot Study	2 (Human)	C	Fracture experience within 3 years	17	72.5 ± 5.2	Without fracture	16	73.8 ± 6.0	
Lecchi *et al.* (2018)	Circulating miR-23b-3p, miR-145-5p and miR-200b-3p are potential biomarkers to monitor acute pain associated with laminitis in horses	1a (Horse)	CC	acute laminitis with no prior treatment various breeds post treatment	9	9.4 ± 5.0 (4 and 17 years)	1)acute laminitis with no prior pre-treatment 2)Control healthy stallions	1)pre(9.4 ± 5.0) 2)Control healthy stallions (2.4 ± 1)	9	N
Mach *et al.* (2016)	Integrated mRNA and miRNA expression profiling in blood reveals candidate biomarkers associated with endurance exercise in the horse	1b (Horse)	QE	pure-breed or half-breed Arabian horses competing in three 160 km endurance competitions	1)Exp set = 14 afterwards (T1) 2)Validation set = 31	10 ± 2	before competitions (T0, baseline)	1)Exp set N = 14 (T0) 2)Validation set = 16	10 ± 2	NA
Mach *et al.* (2017)	Understanding the response to endurance exercise using a systems biology approach: combining blood metabolomics, transcriptomics and miRNomics in horses	1b (Horse)	QE	horses after a 160 km endurance competition	1)Exp set = 10 afterwards (T1) 2)Validation set = 18	9.7 ± 1.5	horses before competition	1)Exp set N = 10 (T0) 1) 2)Validation set N = 13	9.7 ± 1.5	NA
Mandourah *et al.* (2018)	Circulating microRNAs as	2 (Human)	CS	osteopaenia without fracture	131	65.6 ± 9.5	healthy volunteers	30	67 ± 9.6)70 ± 10	N

	potential diagnostic biomarkers for osteoporosis			osteopaenia with fractures OP without fracture OP with fracture		67 ± 9.5 68.6 ± 10 70 ± 10				
McGivney *et al.* (2017)	Evaluation of microRNA expression in plasma and skeletal muscle of thoroughbred racehorses in training	1b (Horse)	QE	after exercise in Thoroughbreds	1)Ci-miRNA = 20 2)Skeletal muscle = 11 training session	1)3 years 2)2 years	Before exercise in Thoroughbreds	1)Ci-miRNA = 20 2)Skeletal muscle = 11 training session	1)3 years 2)2 years	
Panach *et al.* (2015)	Serum circulating microRNAs as biomarkers of osteoporotic fracture	2 (Human)	CC	women with OP subcapital hip fracture	8	63.4 ± 8.1	women with severe osteoarthritis of the hip that required the implantation of a hip prosthesis (controls)	5	79.6 ± 3.1	N
Sansoni *et al.* (2018)	Effects of repeated sprints training on fracture risk-associated miRNA	1b (Human)	RCT	healthy, male adults assigned to EXP group	9	24.3 ± 3.7	healthy, physically active male adults	9	Age matched 23.8 ± 4.4	Y
Seeliger *et al.* (2014)	Five freely circulating miRNAs and bone tissue miRNAs are associated with osteoporotic fractures	2 (Human)	CS	osteoporotic samples	10 30 for a validation	79.3 (66–89)	Nonosteoporotic Sample	10 30 for a validation	78.8 (67–91)	NA/N

Stefaniuk-Szmuki er *et al* (2018)	Transcriptomic hallmarks of bone remodelling revealed by RNA-Seq profiling in blood of Arabian horses during racing training regime	1b (Horse)	QE	GII; after a 24-week period including 12 weeks of light work and 12 weeks sequences of heavy canters	4	2.5 years	G1; before the introduction to the race track	6	2.5 years	N
Stefaniuk-Szmuki er *et al* (2019)	The expression profile of genes involved in osteoclastogenesis detected in whole blood of Arabian horses during 3 years of competing at race track	1b (Horse)	QE	each points of year of training performed divided 10 groups	23 Arabian horses	2.5 years old	NA	NA	NA	N
Sun *et al.* (2020)	Circulating microRNA-19b identified from osteoporotic vertebral compression fracture patients increase bone formation	2 (Human)	CC	osteoporotic patients with or without vertebral compression fractures	Osteoporotic patients with fracture 6/ OP without fracture 6	69.7 (57–80) 68.0 (43–80)	control candidates	6	47.8 (36–59)	N
Turlo *et al.* (2019)	Revisiting predictive biomarkers of musculoskeletal injury in thoroughbred racehorses: longitudinal study in polish population	1a (Horse)	C	Injured Polish Thoroughbred racehorses during longitudinal training	8	2	Control group (no injury)	18	2	N

Varley et al. (2018)	The association of novel polymorphisms with stress fracture injury in Elite Athletes: Further insights from the SFEA cohort	1c (Human)	CC	athletes with stree fracture cohort	125	27.7 ± 7.5	Non-stress fracture	376	24.4 ± 5.4	N
Watts *et al.* (2017)	MicroRNA29a treatment improves early tendon injury	1a (Horse)	RCT	Quarter-Horse-type horses collagenase-induced lesions with miR29a mimic (A)	9	2 to 6 years	Quarter-Horse-type horses Collagenase-induced lesions with placebo-treated (B)	8	2 to 6years	Y
Weilner *et al.* (2015)	Differentially circulating miRNAs after recent osteoporotic fractures can influence osteogenic differentiation	2 (Human)	CS	P-MP F with fractures at the femoral neck	7<br>Validation 12	72.4 ±3.2<br>77.8 ± 1.4	P-MP F without fracture	7<br>Validation 11	71.0 ± 2.3<br>81.5 ± 1.5	N
Yanovich *et al.* (2012)	Candidate gene analysis in Israeli soldiers with stress fractures	2 (Human)	CC	Israeli young active duty soldiers with SF	182	20.1 ± 1.7	without SF	203	20.2±1.3	N
Yavropoulou *et al.* (2016)	Expression of microRNAs that regulate bone turnover in the serum of postmenopausal women with low	1a (Horses)	CS	P-MP women with osteopenia/OP in the lumbar spine and/or total hip or femoral neck	70 (Without VF 35 With VF 35)	68 ± 7<br>71 ± 7	Healthy with normal BMD and no history or X-ray evidence of fracture	35	68 ± 5	N

	bone mass and vertebral fractures			with fragility vertebral fracture (VF) or without VF						
Zarecki et al. (2020)	Serum microRNAs as novel biomarkers for osteoporotic vertebral fractures	1c (Human)	CS	P-MP women with low BMD without VF<br>with VF and low BMD without a treatment<br>with VF and low BMD receiving a treatment for OP	35<br>24<br>17	67.9 ± 5.4<br>69.6 ± 7.0<br>69.6 ± 6.4	P-MP women healthy	42	68.8 ± 6.5	N
Zavodovskaya *et al.* (2018)	Bone formation transcripts dominate the differential gene expression profile in an equine osteoporotic condition associated with pulmonary silicosis	2 (Human)	CS	silicate associated osteoporosis (SAO)-affected	8 Mixed breeds	20 years	unaffected horses	8 mixed breeds	17.5 years	N

Subcategorised topics.1a miRNAs/protein analysis/ gene expression related to stress fractures or biomarkers for musculoskeletal injuries in horses; 1b microRNAs and their targets in response to exercise and mechanical loading in horses and human1c Genome wide association studies related to stress fractures in young adults (e.g. athletes/military recruits) equivalent to equine stress fracture; 2 miRNAs related to osteoporotic fragility fracture in humans; QE; Quasi-Experimental Studies, RCT; Randomised controlled trial, CS; Cross Sectional Study, CC; Case Control, C; Cohort study.

Table S7. Detailed data extraction spreadsheet of selected original research paper—Inclusion and exclusion criteria

Author	Objectives	Country	Inclusion criteria	Exclusion criteria
Arai *et al.* (2008)	To investigate cartilage oligomeric matrix protein (COMP) levels in synovial fluid (SF), serum and urine and the development of osteochondral damage and osteophyte (OP) formation following intraarticular fractures of the carpus	Japan	ascertained arthroscopically and radiographically<br>All of the horses were determined to be free of any<br>other diseases including renal failure and uropathy, based on preoperative assessment of blood and urine	other disease
Arens *et al.* (2013)	To define scintigraphic, physical examination, and scapular ultrasonographic findings consistent with bone fragility syndrome (BFS) in horses; develop indices of BFS severity; and assess accuracy of physical examination, scapular ultrasonography, and serum biomarkers for BFS diagnosis	USA	horses underwent scintigraphy as part of diagnostic<br>evaluation of forelimb lameness	Age (< 5 years) was used as an exclusion criterion for further evaluation
Blott *et al.* (2014)	To identify candidate genome regions associated with<br>fracture risk in the Thoroughbred horse by carrying out<br>a genome-wide association study (GWAS) with 43,417 SNPs genotyped on	UK	sustained catastrophic distal limb fractures (knee and hock)<br>No cases with fractures in other bones<br>Control horses with over 4 years of age, racing during the same time period as the cases	other sites of bone fractures (pelvis, neck or skull)
Cappelli *et al.* (2018)	To identify serum profile of miRNAs in response to prolonged endurance exercise in samples obtained from four competitive Arabian horses before and 2 h after the end of competition	Italy	T0 before competition<br>VS<br>2 h T1 after the end of competition	NA
Chatzipapas *et al.* (2009)	To evaluate the association between VDR polymorphisms and calcaneal Stiffness Index (SI) with stress fractures in a case control study including male military personnel	Greece	stress fracture was diagnosed with an anterior/posterior and lateral radiograph, or a technetium-99 bone scanning	only male military personnel beyond basic training included
Cleary *et al.* (2010)	To investigate the effects of exercise and osteochondral injury on concentrations	USA	All horses were considered clinically normal and without MCP or carpal joint injury on the	NA

	of carboxy-terminal telopeptide fragments of type II collagen (CTX-II) in synovial fluid (SF) and serum of Thoroughbred racehorses and to compare findings with radiographic and arthroscopic scores of joint injury severity		basis of clinical and radiographic examination<br>Findings in the beginning of the study.<br>underwent a similar race-training program<br>Osteochondral injury group undergoing arthroscopic surgery for removal of osteochondral fragments attributable to training or racing injuries.	
Desjardin et al (2014)	To evaluate the role of miRNAs in the regulation of mechano-transduction pathways, samples were also subjected or not to an experimental mechanical loading.	France	Healthy samples and OC predisposed samples were subjected or not to an experimental mechanical loading.<br>1) Healthy VS OC<br>2) Healthy unloaded VS Healthy loaded<br>Healthy loaded VS OC loaded	NA
Farries *et al.* (2019)	To identify heritable variation in the Thoroughbred skeletal muscle transcriptional response to exercise and training	Ireland	Different time points rest and after high-intensity exercise	NA
Feichtinger *et al.* (2018)	To innvestigate the association between serum-levels of 19 miRNA biomarkers of idiopathic osteoporosis to bone microstructure and bone histomorphometry based upon bone biopsies and μCT	Austria	idiopathic osteoporosis	secondary causes for osteoporosis including diabetes mellitus type 1 and 2, inflammatory diseases other disease
Feurer *et al.* (2019)	To examine the serum levels of 32 preselected miRNAs with reported function in bone and their association with osteoporotic fracture from the OFELY Cohort	France	medical history, and ascertained with medical records or spine X-rays for vertebral fractures	fractures of the head, toes, and fingers excluded
Friedman *et al.* (2014)	To assess the association of intragenic sequence variants in a gene-naive manner, using whole-exome capture and massive parallel sequencing in Israeli Defense Forces (IDF) combat soldiers using a twotires discovery-validation, case–control study design	Israel	SF VS NON-SF	There were no statistically significant differences in anthropometric measures, physical activity habits prior to enrolment, smoking habits and alcohol consumption between the two study groups

Frisbie *et al.* (2010)	To assess 7 serum biomarkers for the detection of musculoskeletal injuries	USA	Horses with solitary musculoskeletal injuries and completion of >2 months	lack of training for >30 days, or completion of 10 study months
Hakansson *et al.* (2018)	To identify RNA-biomarkers that can distinguish between different exercise regimes and that entail clues about muscle repair and recovery after prolonged exhaustive endurance exercise	Norway	1) After exhaustive exercise (EEE) VS before and non-exhaustive endurance exercise elite athletes 2) EEE VS PAD 3) TT60 (endurance exercise) over time VS PAD Inclusion criteria for the biobank were a minimum age of 18 years and lower limb amputation, excluding ankle, foot, or toe amputations.	confirmed or suspected malignancy and inability to give informed consent
Horak *et al.* (2018)	To identify the change of selected extracellular miRNA levels is predictive of good or bad response to specific training programs: explosive strength (EXPL), hypertrophic strength training (HYP) and high-intensity interval training (HIIT)	Czech Republic	EXPL, HYP and HIIT After exercise week 5, 8 vs Week 0	NA
Jackson *et al.* (2009)	To determine whether bone biomarkers (osteocalcin, PICP, ICTP and CTX-I) could be used to identify 2- and 3-year-olds at increased risk of fracture in the subsequent flat racing season	UK	Only cases of fracture that were confirmed by radiography and/or nuclear scintigraphy were included	Fractures that occurred as a direct result of trauma (e.g. kick or fall) were excluded
Jackson *et al.* (2015)	To compare serum concentrations of biomarkers of cartilage and bone metabolism between racehorses with a carpal or metacarpophalangeal or metatarsophalangeal (ie, fetlock) joint injury and matched uninjured control horses	UK	a carpal or fetlock joint injury diagnosed by a veterinarian and their trainer agreed to participate	Subsequently, blood samples collected at monthly intervals from each pair of case-control horses until the case or control horse was no longer available or the control horse became injured
Kim *et al.* (2018)	To identify changes in the blood profiles of microRNAs (miRNAs) and mRNAs induced by exercise in horse leukocytes	Korea	T0 before exercise VS T1 after exercise	NA

Kocijan *et al.* (2016)	To evaluate circulating miRNA signatures in male and female subjects with idiopathic and postmenopausal osteoporotic low-traumatic fractures	Austria	idiopathic osteoporosis preMP women or man under 50 years with low-traumatic fractures or postMP women Healthy matched controls	Secondary osteoporosis
Korvala *et al.* (2010)	To search for possible genetic factors predisposing military conscripts to femoral neck stress fractures	Finland	All military conscripts who had suffered from femoral neck stress fractures and had been treated at the Finnish Defence Forces' military hospitals from 1970 to 1995 were invited to participate in a follow-up examination in 2002 or 2003	NA
Kuemmerle *et al.* (2016)	To discriminate between cell types, tendon-specific genes need to be identified	Switzer-land	All horses were free from any previous history of orthopaedic disease and were slaughtered for reasons unrelated to this study	NA
Ladang *et al.* (2019)	To investigate the ability of an miRNA panel (namely, the OsteomiR° score) to predict fragility fracture risk in older people	Belgium	Participated in a long-term prospective study called the SarcoPhAge study (for Sarcopenia and Physical impairment with advancing Age).	Renal failure
Lecchi *et al.* (2018)	To determine whether miRNAs can be used as biomarkers for acute pain in horses (Equus caballus) affected by laminitis; integrate miRNAs to their target genes and to categorise target genes for biological processes	Italy	pre-treatment VS 7 days after routine laminitis treatment ('post-treatment')	NA
Mach *et al.* (2016)	To perform an integrated analysis of the blood transcriptome and miRNome (using microarrays) in the horse before and after a 160 km endurance competition	France	T0 before competition VS T1 after the end of competition within 30 min	8 horses excluded due to poor metabolic condition or lameness after competition
Mach *et al.* (2017)	To identifiy the relationships between blood metabolome, transcriptome and miRNome, which are specifically regulated by endurance exercise in horses	France	T0 before competition VS T1 after the end of competition within 30 min	ten horses failed a vet gate check for lameness, metabolic disorders or tiredness
Mandourah *et al.* (2018)	To identify circulatory microRNAs associated with osteoporosis using	UK	patients who were over 18 years old and able to consent on their own, and either suffering	all participants who were under 18 years old or unable to

	advanced PCR arrays initially and the identified differentially-expressed microRNAs were validated in clinical samples using RT-qPCR		osteopaenia or osteoporosis or healthy volunteers as a control group	consent on their own or suffering from a disease unrelated to osteoporosis
McGivney *et al.* (2017)	To identify plasma ci-miRNA profiles and skeletal muscle miRNAs before and after exercise in Thoroughbreds (Tb), and to evaluate for the presence and effect of haemolysis on plasma ci-miRNA determination	Ireland	Exercise vs control<br><br>the same stable and were managed and trained in a similar way	MiRNAs with >80% missing data removed from the analysis (7/20 horses from Plasma ci-miRNA).
Panach *et al.* (2015)	To identify specific miRNAs in osteoporotic BF patients compared to those in osteoarthritic controls.	Spain	subjects included women with osteoporotic subcapital hip fracture (cases) and women with severe osteoarthritis of the hip that required the implantation of a hip prosthesis (controls).	fractures due to high-energy trauma, those who had used any medication known to alter bone metabolism, those with cancer, or those under 50 years of age
Sansoni *et al.* (2018)	To determine the effect of a 8-week repeated-sprint on circulating levels of fracture risk-associated miRNA.	Italy	18 x 15 m sprints with 17 s of passive recovery, 3 times/week, over 8 weeks<br>1)Ctrol - T0, T1(4 weeks), T2(8 weeks)<br>2)Exp T0, T1(4 weeks), T2(8 weeks)	Other disease
Seeliger *et al.* (2014)	To identify specific miRNAs in patients with osteoporotic fractures compared with nonosteoporotic fractures.	Germany	Patients were recruited if the following criteria were met: femoral neck or pertrochanteric fracture, indication of a surgical treatment	malignancy; benign ovarian cysts except endometrioma; inflammation; known chronic, systemic, metabolic endocrine diseases including polycystic ovarian syndrome
Stefaniuk-Szmuki er *et al* (2018)	To identify by RNA-Seq method, the possible skeletal system originating transcriptomic profile in peripheral blood of Arabian horses undergoing race training.	Poland	Before and after training (They were raised in the same farm and fed in comparable ways including feeding with high quality hay and oats)	Low quality reads were eliminated, and 94% of high-quality filtrated reads aligned to the reference genome
Stefaniuk-Szmuki er et al (2019)	To evaluate the expression pattern of osteoclastogenic related genes, in whole blood of Arabian horses	Switzerland	Horses were introduced to the race track training when were 2,5 years old, and participated in races in 3 consecutive racing seasons	NA

	during 3 racing seasons in 3 consecutive years			
Sun *et al.* (2020)	To investigate miRNAs in the plasma of osteoporotic patients with or without vertebral fractures compared with nonosteoporotic healthy control subjects	China	osteoporosis patients (T-score ≤ −2.5); osteoporotic vertebral compression fracture patients (T-score ≤ −2.5 with brittle fracture), and control candidates (T-score ≥ −1.0)	currently taking any treatment with influence on the bone turnover or tumours, known chronic, systemic, metabolic, or endocrine diseases including polycystic ovarian syndrome; inflammation; or acute trauma
Turlo *et al*. (2019)	To re-evaluate usability of the predictive serum biomarkers identified in North American Thoroughbred racehorses in a geographically distinct group of Polish Thoroughbreds.	Poland	Eighteen horses completed the whole study period without manifesting clinical injury, 6 horses were diagnosed with musculoskeletal injury in the third month of the study and 2 horses sustained injury shortly after the end of the study.	2 horses left the racetrack before completion of 3 study months due to weak performance unrelated to lameness
Varley *et al*. (2018)	To determine, in conjunction with a wider investigation, whether 11 genetic variants in the vicinity of vitamin D, collagen and Wnt signalling pathways were associated with stress fracture injury	UK	Elite athlete with previous stress fracture<br>Vs non-stress fracture	NA
Watts *et al*. (2017)	To evaluate the potential of a miR29a replacement therapy as a therapeutic option to treat tendinopathy in an equine model	USA	Two randomly assigned groups miR29a or Control<br>1)miR-29 treatment group (miR29a mimic)<br>2)placebo-treated tendons<br>Other than an on-site control officer, all investigators were blinded to treatment group.	NA
Weilner *et al*. (2015)	To test whether circulating miRNAs that exhibit changes in recent osteoporotic fracture patients could be causally related to bone metabolism	Austria	P-MP female postmenopausal recently sustained OP fractures at the femoral neck > 65 years old<br>All white Caucasian descent	undergoing chronic treatment with substances known to affect bone metabolism such as anti-resorptive or bone anabolic drugs or glucocorticoids, pathologic or high-trauma fractures
Zarecki *et al*. (2020)	To compare the levels of circulating miRNAs in older women with osteoporotic vertebral fractures, and/or low BMD and healthy controls, and to correlate	Austria	P-MP women, healthy or with low BMD/without fractures or low BMD with VF or with low BMD<br>without a treatment against OP or with low BMD with	One subject based on the data quality check, eight subjects due to visible haemolysis, one subject due to the lack of clinical

	miRNAs expression levels with BTMs		VF with a treatment for OP	information about medications
Zavodovskaya *et al.* (2018)	To examine SAO affected and unaffected horses histologically and subjected to RNA sequencing	USA	control horses included absence of exposure to SAO endemic regions absence of granulomatous pulmonary disease on post-mortem examination.	Horses treated with bisphosphonates (zoledronic acid, clodronate and tiludronate) within the past twelve months excluded
Yanovich *et al.* (2012)	To investigate the association of polymorphisms within candidate genes which we hypothesized may contribute to stress fracture predisposition	Israel	clinically evaluated by an orthopedic surgeon whose physical examination focused on the lower limbs. Imaging included Technetium-99m methylene diphosphonate (Tc99) bone scan	Individuals with grade 1 or a single grade 2 SF by the stated criteria for SF diagnosis and grading as well as individuals with metatarsal SF excluded
Yavropoulou *et al.* (2016)	Tinvestigate serum levels of speci!c microRNAs, known regulators of bone metabolism, in postmenopausal women with low bone mass and with or without vertebral fractures (VFs)	Greece	P-MP women with osteopenia/OP in the lumbar spine and/or total hip or femoral neck with fracture or without fractures	history or presence of bone disease other than primary osteoporosis medication known to affect bone metabolism

Table S8. Detailed data extraction spreadsheet of selected original research paper—Data on sex, sample source and case/control type.

Author	Gender (F, M)	Consent/ ethical approvement	Sample type	Sample collection methods	Case/control Diagnosis
Arai *et al*. (2008)	NA	owner's consent form	SF, Serum, and Urine	stored at –70°C	ascertained arthroscopically and radiographically
Arens *et al*. (2013)	19, 29	owner's consent form	Blood collected from between 8:30 am and 10:30 am	Serum biomarkers	Fracture cases were confirmed by Scintigraphy, Physical examination and severity index, Ultrasonography
Blott *et al*. (2014)	Case (62,207)<br>Control (33, 220)	written consent, Y	bone marrow biopsies (cases) or blood samples (controls)	DNA from bone marrow or EDTA blood (aliquots; a concentration of 70 ng/ul)	Post-mortem
Cappelli *et al*. (2018)	3, 1	written consent, Y	Blood/plasma	Plasma collected, centrifuged with 3,000 × g for 5min	All horses in the same training stable and subjected to the same management practices at the stable and throughout the ride
Chatzipapas *et al*. (2009)	All male	written consent, Y	Blood	Genomic DNA	radiograph or a technetium-99 bone scanning
Cleary *et al*. (2010)	NA	owner's consent form	blood samples, SF samples	stored at –80°C	radiographic and arthroscopic examinations
Desjardin *et al*. (2014)	OC from male<br>Healthy mare<br>But progeny Not known	written consent, Y	Cartilage<br>Bone	After compression, tissues frozen at -80	Clinical examination<br>x-ray<br>OC further confirmed by histology
Farries *et al*. (2019)	64, 47	owner's consent form	Muscle biopsy	DNA	NA
Feichtinger *et al*. (2018)	Female groups<br>Male group	written consent, Y	Blood/serum<br>Bone biopsy	RNA from blood/serum bone	Vertebral fractures assessed by lateral and antero-posterior digital X-rays of the lumbar and thoracic spine. Peripheral fractures evaluated by questionnaire
Feurer *et al*. (2019)	All F	written consent, Y	Blood collected between 8:00 a.m. and 9:30 a.m. after an overnight fast	Serums frozen at -80 °C	Fracture defined based on medical history, and ascertained with medical records or spine X-rays for vertebral fractures, BMD measured by DXA
Friedman *et al*. (2014)	All M	written consent, Y	Blood	DNA extraction from blood	clinical examination and bone scan

Frisbie *et al*. (2010)	51, 79	owner's consent form	blood in serum and sodium citrate tubes, centrifuged at 3000 g for 10 min to obtain serum or plasma fraction Monthly longitudinal samples	Serum or plasma at -20°C	Injuries definitively diagnosed using the diagnostics (clinical, radiographic and scintigraphic examinations available)
Hakansson *et al*. (2018)	EEE, TT60 all male PAD 16 (87.5% male)	written consent, Y	Blood, EDTA tubes, Plasma 2,500 xg, 10 min at 4^{0}C stored at - 80 ^{0}C until analysis	Blood, RNA	EEE, TT60; all subjects had competitive experience at national or international level and trained PAD - All patients were scheduled for lower limb amputation as a consequence of critical limb ischemia.
Horak *et al*. (2018)	All M	written consent, Y	Blood	Plasma from blood	anthropometric parameters
Kuemmerle *et al*. (2016)	3, 3	owner's consent form	harvested tissue in RNAlater and stored at −80 °C,	tendon from the forelimb region of the SDFT, cancellous bone, cartilage, cervical dermal tissue	NA
Jackson *et al*. (2009)	2-year-olds (231/298) 3-year-olds (114/211)	owner's consent form	Blood samples, before exercise between 06.00 h and 08.00 h	Serum separated within 90 min, and stored at -80ºC	Fracture cases were identified by making regular visits, radiography and/or nuclear scintigraphy
Jackson *et al*. (2015)	NA (matched for age, sex, and trainer)	Trainer/owner's agreement	blood samples as soon as possible after the diagnosis of an injury, subsequently, blood samples collected at monthly intervals from each pair of case-control horses	serum separated and stored at –80°C	a carpal or fetlock joint injury diagnosed by a veterinarian and their trainer
Kim *et al*. (2018)	All M	written consent, Y	blood	samples were centrifuged at 4oC at 1,000 × g for 10 min	NA
Kocijan *et al*. *(2016*)	43, 32	written consent, Y	Bone biopsies, serum for miRNA, blood analyses, and clinical investigations	RNA from serum samples	Lateral and antero-posterior digital x-rays of the thoracic and lumbar spine to

					diagnose vertebral fractures. peripheral fractures self-reported by questionnaire
Korvala *et al.* (2010)	All male	written consent, Y	blood samples	Genomic DNA	diagnosis of stress fracture was originally based on accepted radiographic, scintigraphic, or MRI criteria
Ladang *et al.* (2019)	Fracture (16, 1) Control (14, 2)	written consent, Y	Serum	Serum collected at study enrolment	Fractures were self-reported, BMD was measured 3 years after entering the study with DXA
Lecchi *et al.* (2018)	All M	written consent, Y	Serum	Blood collected and serum stored at −80°C	equine veterinarian after physical examination and behavioural evaluation
Mach *et al.* (2016)	20, 41	written consent, Y	Serum for biochemical assays Whole blood for transcriptome and miRNome profiling	collected in PAXgene Blood RNA tubes	horses subject to the same management practices throughout the ride the animals underwent veterinary checks every 20 to 40 km
Mach *et al.* (2017)	13, 28	written consent, Y	Serum for biochemical assays Whole blood for transcriptome and miRNome profiling	collected in PAXgene Blood RNA tubes	veterinary checks
Mandourah *et al.* (2018)	Case (53/10) (13/2) (28/6) (17/2) Control (20/10)	written consent, Y	serum and plasma samples	frozen at −80 °C, RNA, miRNAs	Based on the Bone Mineral Density (BMD) and the *T*-Score, participants were classified into 5 sub-groups
McGivney *et al.* (2017)	1) Ci-miRNAs (10, 10) 2)Skeletal muscle miRNA (4, 7)	written consent, Y	Blood (ci-miRNA) Muscle	blood samples collected in EDTA tubes skeletal muscle from the gluteus medius using a 6 mm-diameter, modified Bergstrom biopsy needle	All horses from the same stable and managed and trained in a similar way. Horses trained 6 days per week with gradual introduction of 800–1000 m sprint training alternating with submaximal training, following which horses entered competitive racing

Panach *et al.* (2015)	All F	written consent, Y	blood sample	Serum separated and stored at -80 °C	DXA
Sansoni *et al.* (2018)	All M	written consent, Y	Blood samples RS 3 times/week T0 and after 4 (T1) and 8 weeks (T2), plasma and serum by centrifuging at 1300 g for 10 min at 15° C	Blood collected after overnight fasting, between 8:00 and 9:00 in K2EDTA spray-coated	no history of other diseases, no traumatic fractures in 2 years
Seeliger *et al.* (2014)	Osteoporotic (7, 3) Non-osteoporotic (0,10) validation serum samples (all F) bone tissue samples (29, 11)	written consent, Y	blood and bone tissue (during the implantation)	Serum Not specified	based on clinical, radiographic and dual-energy X-ray absorptiometry (DXA) evaluation.
Stefaniuk-Szmuki er *et al.* (2018)	NA	owner's agreement	blood samples collected by jugular venipuncture into a Blood RNA Tube and stored at −20 °C until analysis	blood samples, RNA	NA
Stefaniuk-Szmuki er *et al.* (2019)	NA	owner's agreement	blood samples stored at −20 °C	Blood, RNA	NA
Sun *et al.* (2020)	15, 3	written consent, Y	Blood, plasma	miRNA	NA
Turlo *et al.* (2019)	The final population 26 (11, 15)	sample collections as part of nonexperiment al clinical veterinary practice consented by the trainers and the owners	Blood samples collected in one-month intervals, between 6:00 and 7:00 AM after feeding and before daily exercise	Serum separated for biomarker analysis	Diagnosis was made by equine veterinarians cooperating with the racing stable and made available to the authors of the study
Varley *et al.* (2018)	518 elite athletes (69, 449)	written consent, Y	Saliva	DNA	Self-reported stress fracture history (by confirmed imaging tools)
Watts *et al.* (2017)	(A) miR-29a (5, 4) (B) Control (Placebo) (2, 6)	written consent, Y	superficial digital flexor tendon (SDFT)	tendon lesion snap frozen in liquid nitrogen, pulverized in a freezer mill, and stored at −80 °C.	Clinical/ultrasonography

Weilner *et al.* (2015)	All F	written consent, Y	Blood, subcutaneous adipose tissue obtained during tumescence liposuction for osteogenic differentiation of mesenchymal stem cells in vitro	Serums frozen at −80 °C	BMD by DXA
Yanovich *et al.* (2012)	Case 182 (17/165)<br>Control 203 (41/162)	written consent, Y	Blood	DNA extraction was completed within 4 hours from the blood sample	Bone scan
Yavropoulou *et al.* (2016)	All F	written consent, Y	Blood, serum	miRNA	X-rays, dual-energy X-ray absorptiometry
Zarecki *et al.* (2020)	All F	written consent, Y	Serum	Serum taken after an overnight fast and stored at −80 °C	BMD measured at the lumbar spine in minimum of two unfractured lumbar vertebrae using the Hologic Discovery A densitometer, VF identified on images from DXA-based vertebral fracture assessment
Zavodovskaya *et al.* (2018)	7, 9, Mixed breeds	owner's consent form	The skull, scapulae, humeri, ribs, pelvis, lungs, lymph nodes, endocrine organs, skeletal muscles, major abdominal organs and reproductive organs	Rib bone marrow, lymph nodes for RNA	clinical evaluation included physical examination and complete blood cell count and serum biochemistry including ionized calcium concentrations<br>Sections of ribs and other bones evaluated microscopically to confirm SAO

Table S9. Detailed data extraction spreadsheet of selected original research paper—Methods.

Author	Sample prep (e.g. RNA extraction)	Analytic methods	Normalisation	Statistical test
Arai *et al*. (2008)	COMP Levels in SF, Serum, and Urine	ELISA	NA	Factorial ANOVA, and Scheffé's method
Arens *et al*. (2013)	CTX-1 and bone-specific alkaline phosphatase activity (BAP)	ELISA	NA	Shapiro-Wilk test, Two-way ANOVA and the Wilcoxon rank sum test, Spearman correlation coefficients
Blott *et al*. (2014)	DNA extracted using Nucleon BACC DNA extraction kits	Equine SNP50 BeadChip contains 54,602 SNP assays analysed with GenomeStudio software	NA	Cochran-Mantel-Haenszel (CMH) association test, haplotype logistic regression, estimates of the genetic variance obtained with Restricted Maximum Likelihood (REML) analysis
Cappelli *et al*. (2018)	miRCURY TM RNA Isolation Kit—Biofluids Library Preparation and Next-Generation Sequencing NEBNEXT library generation Illumina NextSeq 500 system, producing 50-nucleotide single end reads kit	Bioinformatic Analysis Differential Gene Expression Analyses (edgeR statistical software package) Target Genes and Enrichment Analysis	Spike-in mix (UniSp2, UniSp4, UniSp5 RNA) Normalization carried out using the trimmed mean of M-values method based on log2-fold changes and absolute gene-wise changes (TMM normalization).	Bioinformatics analysis edgeR statistical software package comparing the two groups applying a GLM procedure (a generalization of a paired t-test). p-values for significantly differentially expressed microRNAs were estimated using an exact test on the negative binomial distribution
Chatzipapas *et al*. (2009)	Blood, DNA	DNA genotyping Four different VDR polymorphisms (FokI in exon 2, BsmI and ApaI in intron 8, and TaqI in exon	NA	Kolmogorov-Smirnov test, chi-square test, Student's t-test, One-way analysis of variance (ANOVA)

		9) using PCR amplification		with Tukey's test, Multivariate stepwise linear and logistic regression models
Cleary *et al.* (2009)	carboxy-terminal telopeptide fragments of type II collagen (CTX-II) immunoassay (ELISA)<br>Arthroscopic and radiographic scores	NA	NA	extreme Studentized deviate tests, a paired t test, unpaired t test, a Fisher exact test, Spearman rank correlation
Desjardin *et al.* (2014)	~150mg of tissue frozen at −80°C in liquid nitrogen.<br>The mirVana™ miRNA Isolation Kit	RNA-seq; SOLiD® Total RNA-Seq Kit (Applied Biosystems, Life Technologies).<br>Validation - Taqman qPCR technology	RNA-seq; negative binomial distribution<br>qPCR; reference genes based on RNA-seq results, hsa-mir- 125a-5p, hsa-mir- 331	RNA-seq; Benjamini and Hochberg method<br>miRNA expression patterns by principal component analysis (PCA)
Farries *et al.* (2019)	muscle biopsies	DNA at rest or after high-intensity exercise<br>mRNAs Profiling study in horse RNA-Seq<br>Genomic association study<br>Genotyping	NA	threshold for significant enrichment was set at < 0.05 after adjustment using the Benjamini-Hochberg procedure
Feichtinger *et al.* (2018)	miRNeasy RNA extraction kit<br>quantitative PCR	osteomiR software (using an in-house script developed to remove technical variance using spike-in controls assays and imputation of missing values).	Spike in controls - UniSp4, cel-miR-39-3p, UniSp3	Quantitative clinical characteristics, bone microarchitecture, and bone histomorphometry were compared between groups (preMP, postMP, males) by overall F-Tests and t-tests for pairwise group comparisons.
Feurer *et al.* (2019)	miRCURY Biofluids RNA Isolation Kit (Exiqon), customdesigned Pick-&-Mix microRNA PCR panel using locked nucleic acid (LNA) probes,	Quantitative PCR<br>2-ΔΔCT	UniSP6	Student t test to assess the cohort for Pre-MP and P-MP, between the different groups of Women; the Wilcoxon test; logistic regression for age adjuestment
Friedman *et al.* (2014)	DNA<br>Next generation sequencing	Initial screened variants filtered by	NA	False discovery

		statistically significant differences, subsequently individually genotyped using the Sequenom assay, The haplotype association test between cases and controls was performed using Haploview 4.2		rate (FDR) (Q value of the R package) adjustment for multiple comparisons followed by the chi-square test of the allele distribution in the DNA pool
Frisbie *et al*. (2010)	CS846, CPII epitopes, GAG, Col CEQ, type I collagen (CTX), type I and II collagens (C1, 2C) using ELISA OC using an EIA kit (enzyme immunoassay)	All outcome variables log transformed (natural log) to meet assumptions of normality	Quantification of GAG by papain digesting biological sample, standard curve	Chi-squared analysis,
Hakansson *et al*. (2018)	miRCURY RNA Isolation Kit miRCURY LNA Universal microRNA PCR, by qPCR using microRNA Ready-to-Use PCR, Serum/Plasma Focus panel using ExiLENT SYBR Green Master Mix microRNA specific Taqman qPCR kits snoRNAs analyses, High-capacity RNA to cDNA kit, quantified by RT-qPCR	All data were normalized to the average of all assays in all samples (average Cq–assay Cq	SnoRNA expression was normalized to U6<br><br>miRNA-191 for miRNA	Grub's test was used to identify significant (a < 0.05) outliers in ncRNA measurements One-way analyses of variance (ANOVA) was used to determine differences in expression levels over time comparing mean expression between different cohorts two-tailed Student's t-test
Horak *et al*. (2018)	Plasma microRNA expression quantification by quantitative PCR	Final concentrations were expressed as the 40-Ct values	NA	Shapiro ± Wilk normality test, non-parametric Friedman test, Differences in other variables; ANOVA, Bonferroni multiple comparison post hoc test, Pearson correlation analysis, Spearman rank correlation analysis
Jackson *et al*. (2009)	Serum OC, Carboxy-terminal propeptide of type I collagen (PICP), Crosslinked	competitive immunoassay, radioimmunoassay	NA	Mann-Whitney U test, Chi-squared

	carboxy-terminal telopeptide of type I collagen (ICTP) and CTX-I			tests, stratified analyses
Jackson *et al.* (2015)	Serum concentrations of 2 cartilage synthesis biomarkers (carboxy-terminal propeptide of type II collagen [CPII] and chondroitin sulfate epitope 846 [CS846]), 2 cartilage degradation biomarkers (neoepitope generated by collagenase cleavage of type II collagen [C2C] and cross-linked carboxyterminal telopeptide fragments of type II collagen [CTX-II]), and serum activity of a bone formation marker (bone-specific alkaline phosphatase [BAP]) were measured around the time of injury diagnosis and monthly thereafter for as long as possible.	NA	NA	Changes in serum GAG and CPII in racehorses at risk of injury appear to be similar across distinct populations while dynamics of serum bone marker is more
Kim *et al.* (2018)	NGS analysis for miRNA transcriptome microarray	Bioinformatical - The normalized microarray expression data were analyzed by using GeneSpring GX12 software	sequences normalized as reads per kilobase million (RPKM)	Benjamini-Hochberg's FDR method
Kocijan *et al.* (2016)	One hundred eighty-seven miRNAs were quantified in serum by qPCR, compared between groups and correlated with established bone turnover markers	Delta Cq used as a relative log2-transformed measure for expression levels	Y, mean Cq-value	two-tailed Mann-WhitneyUtest, Benjamini Hochbergs method for false-discovery rate calculation
Korvala *et al.* (2010)	Blood	Polymerase chain reaction amplification of 51 exons of COL1A1, 52 exons of COL1A2, and 23 exons of LRP5, Genotyping	NA	chi-square test, Haploview software, Fisher's exact probability test
Kuemmerle *et al.* (2016)	Cell isolation and culture, RNA isolation, Illumina RNA Sequencing	Bioinformatics analysis (Trimmomatic, tophat version 2.0.14 and gene-level counting	quantitative PCR Data normalized to 18S	ANOVA) followed by Tukey's post-hoc test for multiple group comparisons

		with HTSeq version 0.6.1, DESeq, GeneGo tool).<br>Validation by quantitative PCR and presented as 2^DDCT.		
Ladang *et al.* (2019)	Serum, miRNA<br>cDNA synthesis OsteomiR test kit (TamiRNA)<br>Exilent SYBR green master mix	Quantitative PCR<br>2- ΔΔCT	Unisp4 values	Shapiro–Wilk test, a Spearman correlation test to investigate the associations, a ROC curve analysis to obtain the most adequate balance between sensitivity and specificity. For comparisons between groups, a Mann–Whitney U Test
Lecchi *et al.* (2018)	miRNeasy Serum/Plasma Kit quantitative PCR using TaqMan® probes.<br>Behavioural recordings Composite Pain Scale(CPS) and facial expression-based pain coding System (HGS)	The area under the receiver operating curve (AUC) was then used to evaluate the diagnostic performance of miRNAs	spike-in cel-miR-39	Kolmogorov–Smirnov<br>Mann–Whitney test<br>Spearman's ρ test for any correlation among the expression levels of the various miRNAs, HGS and CPS
Mach *et al.* (2016)	RNA isolation - PAXgene Blood RNA Kit<br>Equine 4x44 K microarray for transcriptome<br>custom equine miRNA 8 × 60 K microarray for miRNome<br>quantitative RT-qPCR with ABsolute Blue qPCR SYBR Green ROX mix for subset of candidate genes<br>miRCURY LNA™ Universal RT microRNA PCR system	Bioinformatic analysis - - gene ontology (GO) terms and we characterized the transcription factors (TFs)<br>ΔΔCt method	A (SDHA), and beta actin (ACTB) as the endogenous reference genes<br>hsa-miR-191	expression data quantile-normalized<br>Differences revealed by the PCA using the Monte Carlo Permutation Procedure<br>correction for multiple testing using the Benjamini-Hochberg method
Mach *et al.* (2017)	proton nuclear magnetic resonance (1H NMR)-based metabolomic analysis<br>microarrays	pathway analysis on Metpa<br>54 metabolic peaks several amino acids, energy metabolism-related metabolites,	A (SDHA), and beta actin (ACTB) as the endogenous reference genes, hsa-miR-191 for miRNA	regression model<br>Monte Carlo testing<br>ANOVA, Bonferroni corrected<br>corrected for multiple testing using the Benjamini

		saccharides, and organic osmolytes in the plasma		and Hochberg method
Mandourah *et al.* (2018)	Blood -plasma, serum	Human Serum & Plasma miRNA PCR Array - Bioinformatic analysis quantitative PCR using the 2−ΔΔCT method	SNORD96A and RNU6–6P	one-way ANOVA with post hoc 'Bonferroni's multiple comparisons test' and two-tailed Mann Whitney test, Pearson correlation coefficient between relative expression level and lumber spine (L2-L4) T-score
McGivney *et al.* (2017)	ci-miRNA using Trizol an three steps of phenol/chloroform purification TaqMan Array microRNA Assays (Applied Biosystems)	Quality control measures (inter-plate calibration, omission of outliers, miRNAs with Ct values >34 from analysis) were carried out using GeneEx software	Exogenous RNA spike-ins (UniSp2, UniSp4, UniSp5, cel-miR-39-3p)	A paired Student's t-test to compare T0 and T5min. A Shapiro-Wilk test, the Benjamini and Hochberg method used to correct for multiple testing, Pearson's rank correlation used to evaluate correlation between T0 and T5min
Panach *et al.* (2015)	Serum, miRNA miRNeasy Mini kit UniversalcDNA synthesis II kit (Exiqon) miRCURY LNA Universal RT microRNA PCR, Serum/Plasma Focus microRNA PCR Panel	Quantitative PCR 2- ΔΔCT	miR-140-3p and miR-93-5p	False discovery rate (FDR) correction was used for multiple comparisons, Tukey's test, ANOVA
Sansoni *et al.* (2018)	miRCURY™ RNA Isolation Kit miRCURY LNA™ Universal RT microRNA PCR and Universal cDNA synthesis kit II (Exiqon),	Relative miRNA expression by real-time PCR, biochemical markers analyses, Milliplex immuno-based fluorescent Luminex assay on a Human Bone Magnetic Bead Panel avoiding inter-assay variability.	Extraction efficiency (spike-ins: Sp2, Sp4,Sp5) Sp6 and Cel39 as internal controls miR-425-5p and miR-484 as housekeeping Hemolysis checked by calculating the miR-23a-to-miR-451 ΔCT ratio	D'Agostino-Pearson omnibus normality test) one-way ANOVA for paired samples and post hoc Bonferroni's test between-group differences, at each time point, unpaired t test, The correlations by

				Spearman's rank correlation test
Seeliger *et al*. (2014)	Human Serum & Plasma miRNA PCR Array MIHS-106Z (Qiagen)	The expression level of the miRNAs from the array was determined by the cycle number via q-PCR using the 2^DDCT method	SNORD96a and RNU6	Two-tailed Mann-Whitney U test To determine the diagnostic utility of serum miRNA, Receiver operating characteristic (ROC) curves
Stefaniuk-Szmuki er *et al* (2018)	MagMAX™-96 Total RNA isolation kit, cDNA libraries (TruSeq RNA Kit v2), RNA-Seq HiScanSQ platform (Illumina)	Bioinformatics analysis (FastQC software, STAR aligner, RNA-SeQC software, DESeq2, DAVID software, the Kyoto Encyclopedia of Genes and Genomes (KEGG)) qPCR validation	GAPDH	Fisher's exact p-value, Benjamini-Hochberg correction method
Stefaniuk-Szmuki er *et al*. (2019)	MagMAX™—96 Total RNA isolation kit, quantitative PCR	2^DDCT	GAPDH and SDHA	one-way ANOVA with Duncan's post hoc and Tuckey's tests
Sun *et al.* (2020)	Plasma, miRNA	quantitative PCR	U6	One-way analysis of variance (ANOVA, then post hoc tests with Bonferroni correction, ROC
Turlo *et al*. (2019)	Serum concentrations of bone and cartilage biomarkers ELISA kit	NA	NA	Shapiro-Wilk test, unpaired t-test or Mann-Whitney test, two-way interaction for the injured and the control group by time period was performed using mixed model ANOVA
Varley *et al.* (2018)	Saliva samples, genotype determined using a proprietary fluorescence-based competitive allele-specific polymerase chain reaction assay	PCR based using allele-specific forward primers and single reverse primer to identify SNPs	NA	Student's t test, Pearson's chi-squared test was used to assess associations, Benjamini and Hochberg false discovery rate test in order to account

				for multiple comparisons
Watts *et al.* (2017)	freezer mill pulverized tendon samples in Trizol QIAgen mini columns (QIAGEN) miRNeasy kit (QIAGEN) TaqMan mRNA assays (Applied Biosystems) or the miScript primer assay (QIAGEN)	qPCR Histology—H&E, Picrosirius Red (azo dye primarily used in staining for collagen and amyloid)	RNU6-6P (U6B small nuclear RNA) or b-actin	Student's t test, ANOVA, or Mann-Whitney U test
Weilner *et al*. (2015)	miRNeasy isolation kit, Universal cDNA Synthesis Kit II, SYBR Green Mix and LNA-enhanced miRNA primer assays (Exiqon)	Quantitative PCR 2- ΔCT	Normalization of Cp-values based on the average Cp of the miRNA assays detected across all 14 samples (Global mean)	Shapiro–Wilk test, two-sided t-tests and p-values adjusted for multiple testing using Benjamini–Hochberg's method
Zarecki *et al*. (2020)	The miRNeasy Serum/Plasma Kit (Exiqon) Universal cDNA Synthesis Kit II (Exiqon) ExiLENT SYBR Green master mix and LNA-enhanced miRNA primer assays	Quantitative PCR normalized delta Cq values	UniSp4	A twoway ANOVA with Tukey post hoc, relationships between microRNAs (independent variables) and bone turnover markers (dependent variables) were calculated by regression analysis in the IBM SPSS Software version 24
Zavodovskaya *et al.* (2018)	RNeasy Plus Mini Kit, stranded mRNA-Seq Kit, RNA-seq Illumina HiSeq 3000	Bioinformatics analysis (expHTS, STAR v. 2.5.1a aligner, limma-voom pipeline, GO enrichment analysis	NA	ANOVA statistical model that factors in presence of disease (affected/ unaffected), osteoporosis phenotype (mild, severe), tissue type (BM, tLN), and RNA isolation batch
Yanovich *et al.* (2012)	Blood, DNA	DNA extraction SEQUENOM Homogeneous MassEXTEND (hME) Assay SNPs analysis	NA	individual SNP association analyses, were given as odds ratios with accompanying confidence intervals, multiple

				comparisons for all SNPs and for all possible inheritance models, haplotypes constructed using the R haplo.stats software
Yavropoulou *et al.* (2016)	Selection of microRNAs miScript II RT Kit (Qiagen) QuantiTect SYBR Green PCR Master Mix	Quantitative PCR 2- ΔΔCT	two snoRNAs (SNORD95 and SNORD96A) and one snRNA (RNU6-2) used to normalize for variability	Student's t-test of the replicate 2(−Delta Ct) values for each gene in the control group

Table S10. Detailed data extraction spreadsheet of selected original research paper—Results.

Author	miRNAs	Target mRNA/Gene	Validation	Findings
Arai *et al.* (2008)	NA	showed a significant positive correlation between the two different mAbs. There was no significant difference between grade from one to four in the values obtained using SF Horses with OP(+) showed ↑urinary COMP (μg)]/[urinary creatinine (mg)] ratio	NA	Measurement of COMP, especially in urine, has potential as a predictive marker of advanced OA following carpal bone fractures in racehorses
Arens *et al.* *(2013)*	NA	Serum biomarkers were not accurate for BFS diagnosis<br><br>serum bALP concentration was significantly higher for horses with BFS	ROC curve analysis, serum bALP concentration was moderately accurate for BFS diagnosis	None of the tests evaluated were accurate enough to replace scintigraphy for mild disease; physical examination and scapular ultrasonography accurate at moderate to severe BFS
Blott *et al.* (2014)	NA	Significant genetic variation associated with fracture risk on chromosomes 9, 18, 22 and 31. Three SNPs on chromosome 18 and one SNP on chromosome 1 reached genome-wide significance ($p < 0.05$). Two of the SNPs on ECA 18 located in a haplotype block containing the gene zinc finger	NA	Genetic variance explained by SNPs for fracture risk was estimated to be 0.479. Genetic variance estimates for each individual chromosome showed significant variance on chromosomes 9, 18, 22 and 31

		protein 804A (ZNF804A). One haplotype within this block (1.95 times at less risk of fracture than cases); a protective effect, while a second haplotype increases fracture risk (cases at 3.39 times higher).		
Cappelli *et al*. (2018)	↑miR-206, miR-208b, miR-133a miR-1, miR-133b, miR-499-5p, miR-95, miR-224, miR-381 ↓ miR-361-3p, miR-1180, miR-486-3p, miR-504, miR-328, miR-100, miR-296, miR-6529, miR-9177, miR-9021 miR-486-5p	For target analyses, we selected 12 miRNAs, the 6 most downregulated and the 6 most up-regulated miRNAs Muscle remodeling (IGF1R, EGFR, PURB, TAGLN, TMOD2, LASP1, SGCD) Energy metabolism and cellular homeostasis maintenance (various solute carriers, such as SLC5A3, SLC1A2, SLC7A1, STX6. Inflammatory response and modulation of cell migration (IGF1R, EGFR, BCL2, PTMA	NA	miR206, 133a, 133b, 208b, 499-5p, and 486-3p. Major changes were observed in circulating levels of musclespecific miRNAs—collectively called myomiRs which act as modulators of myogenesis, mitochondrial biogenesis, hypertrophy and energy metabolism.
Chatzipapas *et al*. (2009)	NA	The f allele FokI more frequent in patients, while the B allele showed such a tendency. a 2.7-fold and a 2.0-fold increase in risk of stress fractures associated with the f and B alleles (OR, 2.7, 95% CI, 1.2– 5.9; p=0.014 and OR, 2.0, 95% CI, 1.0–4.1; p=0.053). No statistically significant	NA	↓ Tscores associated with the presence of f and B alleles. Mean values of T-scores of SI ↓ in patients

		association at t or a alleles.		
Cleary *et al.* (2010)	NA	CTX-II in SF, CTX-II in SF:CTX-II in serum ratio ↑with joint injuries compared with pre- and postexercise<br>Serum CTX-II in postexercise and injured-horse ↓ than pre-exercise samples.<br>arthroscopic scores were not correlated with SF or serum CTX-II concentration	Sensitivity, specificity, positive and negative predictive values, and likelihood ratio	serum and SF CTX-II concentrations and SF:serum CTX-II ratio, 64% to 93% of serum and SF samples were correctly classified into their appropriate group (pre-exercise, postexercise, or injured-joint samples)
Desjardin *et al* (2014)	1) Healthy VS OC<br><u>Cartilage;</u> 49 differentially expressed (DE) miRNAs (3 ↑, 46 ↓ 5 eca-miR-126-5p, 135a-5p, 451, 486-3p, 486-5p ↓)<br><u>Bone;</u> 41 DE (1 ↓, 40 ↑ 8 eca-miR-1249, 18a-3p, 197, 296-5p, 423-3p, 486-3p, 486-5p, 92a-3p ↑)<br>2) Healthy unloaded VS Healthy loaded<br><u>Cartilage;</u> 19 DE miRNAs (12 ↑, 7 ↓ 2 annotated eca-miR-17-3p ↑, 874-3p ↓)<br><u>Bone;</u> 21 DE miRNAs (9 ↑, 12 ↓ 2 annotated eca-miR-206-3p, 17-3p).<br>3) Healthy loaded VS OC loaded<br><u>Cartilage;</u> 15 DE miRNAs (8 ↑, 7 ↓ eca-miR-874-3p ↑)	About 2400 putative targets identified for miR-126-5p, 135a-5p, 451, 486-3p, 486-5p, miR-1249, 18a-3p, 197-3p, 197-5p, 296-5p, 423-3p, 486-3p, 486-5p, 92a-3p<br>In cartilage, functional annotation of their predicted targets suggests a role in the maintenance of cartilage integrity through the control of cell cycle and differentiation, energy production and metabolism as well as extracellular matrix structure and dynamics. In bone, miRNA predicited targets were associated with osteoblasts and osteoclasts differentiation, though the regulation of energy	by qPCR<br>Healthy VS OC<br><u>Cartilage</u><br>miR-126-5p, miR-486-3p ↓<br><u>Bone</u><br>miR-1249, miR-197 ↑	Identified 609 miRNAs in cartilage<br>622 miRNAs in bone<br>Of these, 300 novel miRNAs.<br>561 miRNAs in both tissues<br>163 miRNA precursor sequence orthologs<br><br>Down-regulation of miRNAs in OC predisposed foals may alter pathways crucial for cartilage maturation, leading to an abnormal extracellular cartilage matrix synthesis.

	Bone; 28 DE miRNAs (24 ↑ 4↓, 2 eca-miR-1-3p ↑, miR-2887↓)	production, vesicle transport and some growth factor signaling pathways.		
Farries *et al.* (2019)	NA	4,992 *cis*-eQTL associated with the expression of 1,922 genes post-exercise; 1,703 *trans*-eQTL associated with 563 genes at rest; and 1,219 *trans*-eQTL associated with 425 genes post-exercise. The gene with the highest *cis*-eQTL association at both time-points was the endosome-associated-trafficking regulator 1 gene (*ENTR1)*, Post-exercise:, a potential role in the transcriptional regulation of the solute carrier family 2 member 1 glucose transporter protein (SLC2A1)	NA	Functional analysis of genes with significant eQTL revealed significant enrichment for cofactor metabolic processes. heritable variation in genomic elements such as regulatory sequences
Feichtinger *et al.* (2018)	miR-29b-3p, miR-324-3p, and miR-550a-3p showed significant correlations to histomorphometric parameters of bone formation as well as microstructure parameters. miR-29b-3p and miR-324-3p ↓ in patients undergoing anti-resorptive therapy	NA	Correlations between miRNAs, Bone Histomorphometry and Microstructure	miR-29b-3p, miR-324-3p, and miR-550a-3p showed significant correlations to histomorphometric parameters of bone formation as well as microstructure parameters. miR-29b-3p and miR-324-p were found to be reduced in patients undergoing anti-resorptive therapy
Feurer *et al.* (2019)	Serum miRNA candidate study None of the miRNAs showed a significant difference by the	OC weakly associated with 21 of the 32 miRNAs other bone remodeling biomarkers (CTX, P1NP, and	NA	No evidence that these 32 preselected miRNAs were not associated with BTMs, BMD, microarchitecture, and or fragility fractures

	menopausal status after adjustment for age. subgroups according to the duration of menopause to low BMD vs normal BMD (OP, non-op, osteopenia) with fracture vs without fracture - no any significant difference after adjustment for age	BAP) not associated with the serum levels of the miRNAs		
Friedman *et al.* (2014)	NA	146 sequence variants 14 sequence variants after FDR 146 candidate sequence variants was used for further individual genotyping NEB, GRK4, SLC6A18, LRRC55, SIGLEC12 and ELFN2 cell development, morphology, survival and death, cell-to-cell signalling and interaction, humoral immune response, inflammatory response, nervous system development and function, and tissue development	Initial validation 15 sequence variants in 15 genes displayed either genotypic or allelic nominal significant different rates (rs7426114, rs4073918, rs3801369, rs716745 and rs3752135) Independent validation phase 2 variants in 2 genes maintained	six variants in six genes (rs7426114, rs2515941, rs4073918, rs716745, rs3752135 and rs2071856) rs4073918 in the SLC6A18 gene consistently displayed two genes (CR1 and OR10H3) harboured two candidate sequence variants each (rs3811381, rs2296160 and rs11670007, rs11670326)
Frisbie *et al*. (2010)	Blood (serum/plasma) Biomarker candidate study in horse CS846, CPII, GAGs, Col CEQ, CTX, type I and II collagens	No significant differences between injury and control at the baseline or entry time point (a trend for IAF horses lower CPII)	NA	The greatest changes appeared to occur 4–6 month prior to injury suggest that longitudinal sampling is critical

	(C1, 2C), OC ELISA, enzyme immunoassay	Monthly longitudinal samples, CS846 and GAG levels ↓ for the IAF and DMD groups compared to control, low CPII for DMD, OC ↓ longitudinally throughout the study, CS846 ↓in IAF 6 months prior to injury<br>CTX ↑ at 7 and one months prior to injury and at one month preinjury than the post injury sample. Similarly, OC ↑ at 6 months preinjury and between 7 and 4 months preinjury		4 biomarkers around 6 and 2 months prior to injury and that there was a significant decrease in articular cartilage biomarkers (GAG, CS486) and an increase in bone biomarker (CTX, OC) levels at these times<br>↑ GAG or ↓ OC with exercise, a normal adaptive response<br>↓ CS846 6 months prior to<br>Injury or until after injury may suggest an impaired synthetic response
Hakansson *et al.* (2018)	1) Control vs EEE<br>miR-193a-5p, miR-29a-3p ↑ response to exercise<br>miR-99b-5p and miR-151a-3p ↓ in the recovery process<br>2nd cycling compared to control<br>(miR-142-3p, miR-29a-3p, miR-141-3p, miR-150-5p, miR-424-5p, let-7g-5p, miR-423-3p ↑), (miR-106b-5p, iR-30d-5p, miR-23a-3p ↓)<br>2) Before exercise elite vs PAD<br>miR-193a-5p ↓(↑ miR-193a-5p , miR-483-5p, miR-2110, miR-155-5p, miR-144-5p, miR-30c-5p, et-7a-5p, miR-122-5p, miR-103a-3p, miR-107 in PAD samples) (↓miR-130a-3p, miR-	miR29a-3p, like the other microRNAs of the miR-29-family, plays a crucial role in the formation and maintenance of the<br>extracellular matrix and controls amongst others, the production of collagens<br>miR-193a interacts with High mobility group box-1 (HMGB1) on angio- and vasculogenesis<br>miR-106b overexpression result in skeletal muscle mitochondrial dysfunction and insulin resistance	3)TT60 (strenuous, but non-exhaustive) VS PAD<br>MiR-29a-3p, miR-193a-5p ↓<br>during 2 hours recovery after the time trial<br>106b-5p and miR-155-5p ↓ after the time trial, but both microRNAs ↑ during recovery<br>myomiR- miR-133b-3p ↓ during 2 hours recovery after the time trial<br>hsa-miR-103a-3p ↑ PAD, ↓ response to	individual microRNA expression difference bigger than between the different time points before, during, and after the 2-day exercise regime<br>miR-29a-3p and miR-193a-5p ↑ miR-29a-3p most consistently regulated<br>miR-29a-3p and miR-193a-5p ↓in response to a 60 min time trial<br>Critical (distinguish between different exercise regimes and indicate the extent of muscle exhaustion in athletes)<br><br>miR-106b-5p<br>recovery after exercise<br>miR-106-5p ↓ after exercise and normalised quickly

	19a-3p, miR-21-5p, miR-19b-3p, miR-148b-3p, miR-101-3p, miR-590-5p, miR-27b-3p, miR-335-5p, miR-106b-5p, miR-495-3p ↑ in response to both exercise sessions, compared to the PAD samples SNORD114.1 ↑over time after the time trial SNORD112, SNORD113.2, SNORD113.6, and SNORD 114.1 differ at TT60 time points or PAD (↑)		exercise PAD ↓ recovery miR-193a-5p, miR155-5p, miR133a-3p, miR-133b-3p, and miR-122-5p ↑in PAD miR29a-3p and miR-495-3p only became different from the PAD cohort after exercise. MiR-29a-3p ↓in the TT60 than in the PAD, directly and at 2 h after the time trial. MiR-495-3p was borderline-significantly ↓	miR-133b-3p and possibly miR-1-3p were regulated in response to exercise after the 60 min time trial. SNORD114.1 ↑in response to exercise miR-29a-3p and miR-495-3p are significantly different in athletes compared to PAD patients (limb ischemia) miR-495-3p could be associated with repair processes that are lacking in patients with severe PAD
Horak *et al.* (2018)	Candidate miRNAs (miR-21, miR-222, miR-16 and miR-93 levels) HIIT miR-21 and miR-93 levels ↑after 5 weeks then ↓to below the initial level after 8 weeks of exercise, while miR-16 gradually ↓ EXPL ↓ in miR-222 after 5 weeks of exercise, which then remained stable	611 verified target genes for miR-16, 377 for miR-21, 1,527 for miR-93 and 1,190 for miR-222	60 verified target genes for miR-16, 113 for miR-21, 35 for miR-93 and 41 for miR-222. Of these, further selected genes linked to exercise-induced physiological adaptations	miR-16, miR-21, miR-222 and miR-93 are down- or upregulated during explosive strength (EXPL), hypertrophic strength training (HYP) and high-intensity interval training (HIIT).

	HYP exercise ↑ in miR-93, miR-16 and miR-222 after 5 weeks of intervention and a subsequent decrease		(i.e. muscle growth, mitochondrial synthesis, angiogenesis, etc)	
Jackson *et al.* (2009)	NA	OC, PICP, ICTP and CTX-I not significantly different between horses that subsequently went on to fracture and those that did not CTX-I concentrations were marginally significantly lower in females that went on to fracture (P = 0.05) comparisons within gender revealed that ICTP and CTX-I concentrations were significantly lower in the 7 female fracture cases compared to the 55 females that did not fracture	NA	bone biomarkers (OC, PICP, ICTP and CTX-I) cannot be used to identify 2- and 3-year-olds that sustain a fracture
Jackson et al. (2015)	NA	CPII, chondroitin sulfate epitope 846(CS846), 2 cartilage degradation biomarkers (collagenase cleavage of type II collagen and CTX-II, and serum activity of a bone formation marker (bone-specific alkaline phosphatase (BAP) by ELISA kits fetlock joint injuries ↓ serum CPII and	ROC curve analysis CPII concentration good sensitivity (82%), low specificity (50%) no convincing evidence of the suitability of biomarkers as diagnostic or prognostic tools	Injured horses lower serum CPII concentrations and significantly higher serum BAP activities than matched control horses. CTX-II were decreased between 2 and 4 months following joint injury. Measurement of CPII at baseline could distinguish betweeninjured horses and control horses

		↑ serum BAP activities than control<br>CTX-II ↓ following joint injury.		
Kim *et al.* (2018)	Exercise-induced DEMs Including 4 known miRNAs and 2 novel miRNAs. ↑miR-423-5p. ↓<br>miR-144, miR-33a, miR-545.<br>Novel miR-14-5p present before exercise but was not detected after exercise. Novel miR-95-3p was not detected pre-exercise but was detected after<br>exercise	potentially targeted 1,625 genes and 905 genes<br>miR-144 targeted 335 genes, miR-33a<br>targeted 353 genes, miR-423-5p targeted 367 genes, miR-545 targeted 570 genes, novel miR-14-5p targeted 115 genes, and novel miR-95-3p targeted 790 genes.<br>13 pathways from the genes targeted by<br>known miRNAs and 16 pathways from the genes targeted by<br>novel miRNAs.<br>DEGs by microarray analysis	10 genes among the candidate 28 genes for physical performance in the horse were identified in the samples<br>of the leukocytes of three Warmblood horses<br>↑ACTN3, IGF1R, AMPD1, DRD1, DRD3, and 5HTT, and<br>↓ HIF1A, GYS1, and VEGFA	The networks of five mRNAs (LOC100050849, LOC100054517, KHDRBS3, LOC100053996,<br>and LOC100062720) and 3 miRNAs (novel miR-95-3p,eca-miR-545, and eca-miR-144) were identified
Kocijan *et al.*(2016)	DE miRNAs were identified in all<br>(46 in preMP, 52 in postMP, 55 in male).<br>19 miRNAs common<br>Eight miRNAs (miR-152-3p, miR-30e-5p, miR-140-5p, miR-324-3p,<br>miR-19b-3p, miR-335-5p, miR-19a-3p, miR-550a-3p) excellent discriminators of patients with low traumatic fractures, regardless of age and sex.	Correlation analysis identified significant correlations between miR-29b-3p and P1NP, and miR-365-5p and iPTH, TRAP5b, P1NP and<br>Osteocalcin, as well as BMDL1–L4 and miR-19b-3p, miR-324-3p, miR-532-5p, and miR-93-5p.	Associations between BTM, a BMD, demographic<br>data and miRNAs; high correlations of four miRNAs (miR-550a-3p, miR-7-5p, miR-378a-5p, and miR-532-5p) to P1NP	Specific serum miRNA profiles are strongly related to bone pathologies. might be directly linked to bone tissue homeostasis.<br>miR-29b-3p has previously reported as regulator of osteogenic differentiation and could serve as a novel marker of bone turnover in osteoporotic patients as a member of a miRNA signature

			Multivariate analysis—miRNA biomarker for osteoporosis miR-942-5p, miR-155-5p, miR-330-3p, miR-203a and miR-181c-5p	
Korvala *et al.* (2010)	NA	COL1A1, COL1A2, OPG, ESR1, VDR, CTR, LRP5, IL-6) interaction between the CTR (rs1801197) and the VDR C-A haplotype was observed	lacking the C allele in CTR and/or the C-A haplotype in VDR higher risk of stress fracture than subjects carrying both (OR = 3.22, 95% CI 1.38-7.49, p = 0.007). LRP5 haplotype A-G-G-C alone and in combination with the VDR haplotype C-A was associated with stress fractures	allele in CTR and the C-A haplotype VDR - a 3-fold higher risk of stress fracture LRP5 haplotype and VDR haplotype associated with stress fractures through reduced body weight and BMI
Kuemmerle *et al*. (2016)	NA	12 genes that were selectively expressed in either tendon (THBS4, TENM4, SCX, ENPEP), ligament (TNMD), bone (BTLN9, CD36, MASP2, SNCG), or cartilage (CHODL, ACAN, THBS3), and two genes that	EYA2 expression levels ↑ in equine tenocyte microtissue spheroids as compared to GPRIN3	eyes absent homolog 2 (EYA2) and a G-protein regulated inducer of neurite outgrowth 3 (GPRIN3) as specific tendon markers compared to bone, cartilage and ligament, equine tendon cells cultured expressed significantly

		were exclusively expressed in tendon (EYA2 and GPRIN3) EYA2 and GPRIN3 as specific markers of equine tendon as compared to ligament, bone and cartilage.	and the selective cartilage marker CHODL	greater levels of EYA2 than GPRIN3, and stained positively for EYA2 using immunohistochemistry. EYA2 found in fibroblast-like cells within the tendon tissue matrix and in cells localized to the vascular endothelium.
Ladang *et al*. (2019)	Serum miRNA candidate study quantitative PCR (OsteomiR test kit) none of the 19 other miRNAs showed a difference a trend of higher OsteomiR° scores in fracture subjects OsteomiR° score ↑ in low BMD	NA	ROC curve analysis AUC= 0.687 Sensitivity 76%, specificity 63%, predictive positive value 68% negative predictive value 71% single miRNA is not sufficient to predict fragility fracture risk	
Lecchi *et al*. (2018)	Detected (hsa-miR-532-3p, hsa-miR-219-5p, mmu-miR-134-5p, mmu-miR-124a-3p, hsa-miR-200b-3p, hsa-miR-146a-5p, hsa-miR-23b-3p, hsa-miR-145-5p and hsa-miR-181a-5p) miR-23b-3p (fold change = 14.6; P = 0.029), miR-145-5p (fold change = 4.4; P = 0.015) and miR-200b-3p	Computational target prediction and functional enrichment identified common biological pathways the glutamatergic pathway affected by miR-23b-3p, miR-145-5p and miR-200b-3p.	miR-23b-3p, miR-145-5p and miR-200b-3p ↑ in pre-treatment the AUCs were 0.854, 0.859 and 0.841	Identified DE miRNAs related laminitis and treatment Both CPS and HGS scores ↓in the control group than in horses with acute laminitis (pre-treatment) Combining two miRNAs in a panel, namely miR-145-5p and miR-200b-3p, increased efficiency in distinguishing animals with acute pain from controls. deregulated miRNAs were positively

	(fold change = 3.4; P = 0.023) in pre-treatment ↑ compared with controls. potential miR-200b-3p ↓ after routine laminitis treatment			correlated to HGS scores.
Mach *et al.* (2016)	cellular miRNAs<br>Total 362 miRNAs found in the whole blood sample, 167 DE miRNAs including 12 equine-specific DEmiRNAs and<br>19 putative novel miRNAs (mitomiR-009 and mitomiR-010 probably<br>encoded by the mitochondrial genome) compared<br>with pre-ride samples<br>91 DEmiRNAs (↑70↓ 21) presented an experimentally annotated targetome , ↓ 7,150 putative target genes<br>miR-138-5p and miR-26b-5p significantly more predicted targets<br>miR-15a, miR-16, miR-17, miR-18a, miR-20ab, miR-21-5p, miR-27a, miR-30b, miR-93, miR-101, miR-106, miR-107, miR-125b, miR-130, miR-138-5p, miR-145, miR-181ab-5p, miR-221, miR-223, miR-342-3p	2,453 DEGs by mRNA microarray<br>↑1,165 associated with the inflammatory response, intestinal permeability and regulation of the response to stress and bacterium<br>↓1,288 at T1 relative to T0<br>Related to macromolecule, catabolism, cellular respiration, mitochondrial transport, and transcriptional and translational activity<br>The main putative regulatory TF; ZFP42, followed by the cooperatively transcriptional cofactors SPI1, FOXO3, IRF3 and NRF1<br>the EP300 protein stimulated transcription<br>of miR-92a, which in turn suppressed EP300 expression	By qPCR<br>miR-181b-5p and miR-505-5p, miR-21-5p; suggesting that it might be involved in the regulation of exercise-related physiological process	44 enriched miRNAs inversely correlated with the expression of 351 target DEGs during exercise<br>RFX5 and<br>FOSL2 both might inhibit expression of miR-192-5p, miR-93-5p and miR-138-5p in blood, although EP300<br>might stimulate expression of miRNAs such as miR-125-5p and miR-223-3p.<br>EP300 might repress miR-92a expression

	and miR-505 previously reports on exhaustive exercise in humans			
Mach *et al.* (2017)	5 miRNAs whose expression was significantly altered at T1<br>107 DEmiRNAs (let-7b-5p, miR-16-5p, miR-21-5p, miR-92a-3p, and miR-192-5p regulated more than ten metabolic genes each)	7,678 DEGs<br>global regulatory network based on 11 unique metabolites, 263 metabolic genes<br>post-competition characterized by lactate, glycerol, creatine, urea, and aromatic amino acids such as tyrosine, along with negative excursion of methylene, N-acetyl moieties, methylene esters, glucose, and phosphocholine<br>glycerolipid metabolism, D-glutamine and D-glutamate metabolism, pyruvate metabolism, tricarboxylic acid and amino acids metabolism<br>FoxO and glucagon signaling pathways to induce gluconeogenesis, and the relationship between lactate and metabolic genes encoding proteins	31 independent animals	31 independent animals, multiple factor analysis confirmed the strong associations<br>between lactate, methylene derivatives, miR-21-5p, miR-16-5p, let-7 family and genes that coded proteins<br>involved in metabolic reactions primarily related to energy, ubiquitin proteasome and lipopolysaccharide immune responses after the endurance competition. Multiple factor analysis also identified potential biomarkers at T0 for an increased likelihood for failure to finish an endurance competition.
Mandourah *et al.* (2018)	Fifteen differentially-expressed microRNAs (over 2-fold changes) were identified between the osteoporosis,<br>female group and the non-osteoporotic, female group	BMP2K, FSHB, IGF1R, PTHLH, RUNX2,<br>SPARC, TSC22D3 and VDR, CYP3A4, MAPK1, CNR2, CYP17A1, CYP19A1, MAPK3 and CNR1	hsa-miR122-5p and hsa-miR4516 are present at significantly different levels between non-	hsa-miR122-5p and hsa-miR4516 in clinical samples also independently showed a strong significant correlation with BMD lumbar spine T-score (Both microRNAs were combined together, there was a much stronger diagnostic

	and 25 up or down (over 2-fold changes) differentially-expressed microRNAs were identified between the osteoporosis, female group and the osteopaenia, female group		osteoporotic control	value for osteoporosis, AUC = 0.75, P = 0.004)
McGivney *et al.* (2017)	Plasma ci-miRNA data for 13/20 horses and all skeletal muscle miRNA data passed quality control. From plasma, 52/179 miRNAs were detected at both time-points. Haemolysis levels were greater than the threshold for accurate quantification of ci-miRNAs in 18/25 resting and all post-exercise plasma samples. Positive correlations (P < 0.05) between haemolysis and miRNA abundance were detected for all but 4 miRNAs, so exercise-induced changes in plasma ci-miRNA expression could not be quantified. Skeletal muscle miRNA, 97/179 miRNAs were detected with 5 miRNAs (miR-21-5p, let-7d-3p, let-7d-5p, miR-30b-5p, miR-30e-5p) differentially expressed	1077 of the predicted targets were unique to these 2 miRNAs while only 3 gene targets were predicted for let-7d-3p. the Jak-STAT signaling, MAPK signaling, insulin signaling and mTOR signaling pathways and long-term potentiation.	Skeletal muscle miRNA by real time PCR miR-21-5p, let-7d-3p, let-7d-5p, miR-30b-5p, miR-30e-5p	Due to the high levels of haemolysis in the plasma samples and the strong positive correlation between haemolysis and miRNA abundance, it was not possible to accurately quantify exercise-induced changes in plasma ci-miRNA expression in the present study. DE miRNAs in skeletal muscle indicates modification of miRNA expression may contribute to adaptive training responses in Tbs. Using a human plasma panel likely limited detection of equine-specific miRNAs.

	(DE, P < 0.05) between time-points.			
Panach et al. (2015)	42 miRNAs DE between groups<br>After correcting with the Benjamini–Hochberg FDR test<br>- ↑miR-144-5p, miR-21-5p, miR-101-3p, miR-210, miR-122-5p, miR-423-5p, and miR-155-5p in fracture group;<br>↑ miR-143-3p, miR-497-5p, miR-125b-5p, miR-365a-3p, and miR-409-3p in the control group	bone metabolism (miR-143-3p, miR-122-5p, miR-125b-5p, miR-210, and miR-21-5p) plus miR-34a-5p since this miRNA appears to be a novel and critical suppressor of bone resorption	miR-122-5p and miR-21-5p indicating their value as biomarkers for distinguishing BF patients from osteoarthritic controls.<br>miR-21-5p, correlated to those of CTx	miR-122-5p, miR-125b-5p, and miR-21-5p
Sansoni *et al.* (2018)	miR-21-5p, miR-23a-3p, miR-24-3p, miR93-5p, miR-100-5p, miR-122-5p, miR-124-3p, miR-125b-5p, miR-148a-3p, miR-637.<br>i) miR-21-5p was stable; ii) miR-23a-3p and miR-24-3p were decreased at T1 and T2 compared to T0 and to CTRL; iii) miR-100 was decreased at T2 compared to T0; iv) miR-122-5p, miR-125-5p, and miR-148-3p were decreased at T1 and then recover to baseline at T2; iv) miR-93-5p was increased by the intervention.	Serum concentrations of bone markers (DKK1, sclerostin, osteoprotegerin, osteocalcin, osteopontin), cytokines (IL-1β, TNFα), and metabolic hormones (leptin, insulin, PTH) were assayed by multiplex assay<br>None of the metabolic hormones was affected by the intervention while, among the bone markers, DKK1, osteocalcin and sclerostin were slightly but significantly decreased.	real-time PCR ROC curves to determine the diagnostic potential of serum miRNAs, which were analyzed after logarithmic transformation of all samples included in the validation	an 8-week repeated-sprint training changed expression of circulating miRNA associated with fracture risk (miR-23a-3p, miR-24-3p were, miR-100, miR-122-5p,<br>miR-125-5p, and miR148a-3p ↓, while miR-93-5p ↑).<br>DKK1, osteocalcin and sclerostin slightly ↓
Seeliger *et al.* (2014)	83 different miRNAs, 51 were detectable in the serum of the	TargetScan Human V5.1 database	miScript SYBR Green PCR Kit with a CFX 96	The associated area under the curve (AUC) was used to confirm the diagnostic value, highest

	osteoporotic patients with hip fractures (serum)<br>11 miRNAs, including miR-21-5p, miR-23-3p, miR-24-3p, miR-25-3p, miR-27a-3p, miR-100-<br>5p, miR-122a-5p, miR-124-3p, miR-125b-5p, miR-148a-3p, and<br>miR-223-3p ↑ in osteoporotic patients (bold -validated) (serum)<br>miR-21, miR-23a, miR-24, miR-25, miR-100, and miR-125b ↑ identified in bone tissue osteoporotic patients	Up to now, for the miRNAs miR-21, miR-23a, miR-24, miR-93, miR-100, and miR-148a, an osteogenic or osteoclastic involvement is known.<br>downregulation of PDCD4 and an<br>upregulation of c-FOS, both influenced by miRNA-21<br>The expression of RUNX2—possibly affected by miRNA-23a/miRNA-24-2/miRNA-27a complex<br>miRNA-93 repressing the gene expression of osterix ↓<br>BMPR2 ↓under the influence of miRNA-100<br>VCAN ↓by miRNA-124a	Touch Real-Time PCR System<br>bone morphogenetic protein receptor type II (BMPR2), c-Fos,<br>osterix (OSX), programmed cell death 4 (PDCD4), receptor activator of NF-kB ligand (RANKL), runt-related transcription factor (RUNX2), and versican (VCAN) by real time PCR	value of 0.77 miR-122a (95% CI 0.69–0.86, p<0.0001), 0.63 for miR-21, miR-23a, miR-24, 0.68 for miR-93, 0.69 for miR-100, miR-124a, 0.76 for miR-125b, 0.61 for miR-148a.
Stefaniuk-Szmuki er *et al* (2018)	NA	97 deregulated pathways including glycerophospholipid metabolism,<br>fructose and mannose metabolism, glycerolipid metabolism, and osteoclast differentiation,<br>twelve genes potentially involved in the metabolism of the skeletal system (BGLAP, CTSK, TYROBP, PDLIM7, SLC9B2, TWSG1, NOTCH2, IL6ST,	The RNA-Seq data agreed with the qRT-PCR data and Pearson correlation coefficient values (PDLIM7, TYROBP, TXLNG, TCAP, CLEC5A, BGLAP)	bone homeostasis was those involved in osteoclast differentiation.<br>Among the significantly expressed molecules, we recognized twelve genes potentially involved in the metabolism of the skeletal system: BGLAP, CTSK, TYROBP, PDLIM7, SLC9B2, TWSG1, NOTCH2, IL6ST, VAV3,<br>NFATc1, CLEC5A, TXLNG. The panel of identified genes should be evaluated as candidate biomarkers for bone

		VAV3, NFATC1, CLEC5A, TXLNG)		homeostasis indicators of Arabians performing on race tracks to assess bone remodelling states during training for race track competitions.
Stefaniuk-Szmuki er *et al*. (2019)	NA	training induced changes in transcript abundance ofgenes (NFATc1, CTSK, DAP12, CLEC5A, IL6ST, VAV3) involved in osteoclastogenesis hence bone resorption	NA	The expression pattern of all analysed genes varied depend of exercise intense Activity, initial training had greater effect on expression pattern
Sun et al. (2020)	total of 338 DEmiRNAs were identified between the osteoporotic group and nonosteoporotic healthy control group 9 miRNAs were significantly downregulated miR-15b-5p, 92b-3p, 374c-3p, 144-5p, 148a-3p, 19b-3p, 106b-5p, 145-3p, 10b-5p)	PTEN (phosphatase and tenesin homolog deleted from chromosome 10) was found to be directly repressed by miR-19b, with a concomitant increase in Runx2 expression and increased phosphorylation of AKT (protein kinase B, PKB).	miR-19b, which was most reproducibly altered in the validation cohort MiR-19b and osteoporosis without vertebral facture had a sensitivity of 91.3%, a specificity of 80.5%, and an AUC of 0.9280 (95% confidence interval [CI] 0.85–0.99, p < 0.0001).	ROC curves of miR-19b and osteoporosis without vertebral facture/ with fracture high sensitivity and specificity miR-19b↑ during osteoblastic differentiation
Turlo *et al*. (2019)	NA	Serum Biomarker candidate study osteocalcin (OC), c-terminal telopeptide of type I	NA	Changes in serum GAG and CPII in racehorses at risk of injury appear to be similar across distinct populations while dynamics of serum bone marker is more

		collagen, total glycosaminoglycans (GAG), chondroitin sulfate epitope and c-propeptide of type II procollagen (CPII)<br>Mean GAG and CPII levels ↓ in injured group comparing to control<br>↓ OC and ↑c-terminal telopeptide of type I collagen (CTX-I) levels in injured horses comparing to controls in polish population		
Varley et al. (2018)	NA	11 SNPs investigated<br>SOST SNP rs1877632 and VDR SNPs rs10735810 and rs731236 associated with stress fracture.<br>In the whole cohort, rs1877632 heterozygotes and homozygotes of the rare allele combined made up 59% of stress fracture sufferers in comparison to 46% in the non-stress fracture group.<br>In the multiple stress fracture cohort, homozygotes of the rare allele of rs10735810 and rs731236 association with stress fracture when compared to those homozygotes for the common allele combined with heterozygotes. No significant associations were	NA	These data suggest an important role for SOST SNP rs1877632 and VDR SNPs rs10735810<br>and rs731236 in the pathophysiology of stress fracture

		shown in the other SNPs analysed		
Watts *et al.* (2017)	miR29a↓ throughout a time course of induced tendon injury (week 3,5, 9,17)	collagen 3(Col3a1) and Col1a1 mRNA ↑ tendon injury miR29a-binding sites conserved in horses<br>miR29a mimic in equine tenocytes selectively regulated Col3a1 early in the model but not Col1a1 mRNA (upregulated significantly less at early time points (13-fold at 3 weeks and 9-fold at 5 weeks, reversed at weeks 9 and 17).<br>miR29a-binding sites are excised from collagen 1 transcripts due to the use of an alternative proximal polyadenylation signal.	qPCR<br>Ultrasound<br>MRI -concurrent tissue molecular phenotype of reduced collagen 3 confirmed macroscopically on T2 mapping, provides convincing evidence that a miR manipulation can target both inflammatory/ matrix crosstalk in tendon disease,	miR29a↓ tendon injury<br>Col3 and Col1 mRNA ↑<br>collagenase-induced tendinopathy in horses and that this inversely correlates with collagen 3 expression. reveals that col1a1 utilizes an alternative poly(A) signal that truncates its 30 UTR with the loss of all three miR29-binding sites.
Weilner *et al*. (2015)	Serum<br>miRNA profiling/candidate study<br>Normalization using global mean of Cp-values across all 14 samples	↑miR-21 (not validated)<br>↓ miR-10a-5p, miR-10b-5p, miR-133b, miR-22-3p, miR-328-3p, let-7g-5p at fracture	5 out of 7 tested miRNAs can modulate osteogenic differentiation of MSCs in vitro	miR-22-3p, miR-328-3p, and let-7g-5p
Yanovich *et al.* (2012)	NA	Assay SNPs analysis within 17 genes<br>25 polymorphisms within 9 genes (NR3C1, ANKH, VDR, ROR2, CALCR, IL6, COL1A2, CBG, and LRP4)	NA	None of the SNP associations remained significant after correcting for multiple comparisons (false discovery rate- FDR).

		showed statistically significant differences<br>17 genetic variants (9 SNPs and 8<br>Haplotypes) were associated with an increased stress fracture risk, and 8 variants (7 SNP and 1 haplotype) with a decreased stress fracture risk.		
Yavropoulou *et al.* (2016)	Serum<br>miRNA candidate study<br>quantitative PCR<br>1)↑ miR-124-3p, miR-2861<br>↓miR-21-5p, miR-23a-3p, miR-29a-3p in OP vs non-OP<br>2) ↓ miR-21-5p in OP with VF Vs OP without VF	miR-21 (SPRY1, DKK2, SMAD7)<br>miR-29 (COL3A1, COL5A3, PTHLH, DUSP2) with regard to osteoblast function<br>MiR-23-3p repressor of Runx2, a key transcription factor of osteoblast differentiation	miR-21-5p AUC 0.66 (95% CI 0.50, 0.81, P = 0.040), sensitivity 66%, specificity 71%<br>miR-29a-3p AUC 0.61 (95% CI 0.45–0.77,<br>P = 0.180)<br>miR-23a-3p AUC 0.63 (95% CI 0.47–0.79,<br>P = 0.115)	
Zarecki et al. (2020)	Serum<br>miRNA candidate study<br>quantitative PCR<br>↑ miR-375, miR-532-3p, miR-19b-3p, miR-152-3p, miR-23a-3p, miR-335-5p, miR-21-5p in PosMP women with VF/low BMD against without VF/low BMD and<br>healthy controls	PINP correlated with miR-451a and miR-188-5p<br>OC correlated with miR-19b-3p,<br>BAP correlated with miR-532-3p, miR-19b-3p,<br>CTX correlated with miR-19b-3p<br>miR-19b-3p; Estroge signaling pathway (ESR1, ADCY1, ATF2, CALM1, PIK3R3, GNAQ PIK3CA)	miR-375, miR-532-3p, miR-19b-3p, miR-152-3p, miR-23a-3p, miR-335-5p, miR-21-5p explain the difference between subjects with and without vertebral fractures by 72.7%	Specific circulating miRNAs reflect the presence of osteoporotic vertebral fractures in postmenopausal Women.<br>No significant differences existed between low BMD and healthy controls.

	miR-486-3p, miR-30e-5p, miR-127-3p, miR-214-3p, miR-550a-3p, miR-106b-5p, miR-133b, miR-143-3p at least one significant ($P < 0.05$)<br>post-hoc test between any of the four group	miR-21-5p; Hippo signaling pathway (YAP1, SMAD7, LATS1, BMPR2)		
Zavodovskaya *et al.* (2018)	NA	osteoporosis phenotype–17 DE genes in BM and 36 DE genes in lymph nodes<br>Bone marrow ↑ *SLC9A2*, *OMD*, *DLX3*, *TRPV4*, *COL24A1*, *SP7*, Osx, *BSP*, BGLAP, *OC*, *SMPD3*, *COLXIA2*, CHAD, *ANO5*, WFDC1, CDH15, *DAPK2*<br>↓*HSD17B6*<br>Lymph nodes ↑*PTX3*, *FCAR*, *DMP1*	Histologically confirmed total bone surface ↑ (osteoblast hypertrophy), bone resorption surface to total bone surface ↑, The lymphoid follicles atrophied, mild to moderate granulomatous inflammation and fibrosis in SAO+ horses	Osteoblasts were hyperplastic and hypertrophic in bone marrow from affected horses.<br>Increased osteoblastic activity - part of the pathological mechanism for osteoporosis or a compensatory of osteolysis

Table S11. Study and participant characteristics for publications included in a systematic review of 20 horse studies. RCT; Randomised controlled trial, QE; Quasi-Experimental Studies, CS; Cross-sectional, CC; Case-control, C; Cohort, F; female, M; male.

Author (Year)	Study design	Study population (Individual, Location, Time)	Sample size (Case/control)	Age (year)	Breed/ Type	Gender (F/M)	Intervention	Outcome	Exclusion criteria (general reason for exclusion) # specific reasons for exclusion in each study
Arai *et al.* (2008)	CS	Equine hospital patient, Japan, NA	51(26/25)	NA	Thoroughbre d	NA	Osteochondral fractures of the carpal joints with osteophyte positive or negative	Synovial fluid, serum and urine peptide biomarkers	Other diseases (e.g. renal failure and uropathy)
Arens *et al*. (2013)	CC	University equine hospital patient, USA, 2008 – 2010	48(20/28)	Case (14, 5 to 25) Control (7, 2 to 16)	various breeds	(19/29)	Bone fragility syndrome or control	Serum bone turnover peptide biomarkers of bone turnover	Age < 5 years
Blott *et al.* (2014)	CC	UK racehorses, UK, 1999 - 2005	522(269/253)	Case (NA) Control (>over 4 years)	Thoroughbre d	(95/427)	Catastrophic distal limb fractures	Genetic association (genetic variation, SNPs)	Fractures in other bones (e.g. pelvis, neck or skull)

Capelli *et al*. (2018)	QE	Horses in training yard	4	6.7	Arabian	(3/1)	Before/after endurance exercise	Serum miRNA profiling	NA
Cleary *et al*. (2010)	CC	University equine hospital patient, USA, NA	78(38/40)	Case (2 to 7) Control (14 to 21 months)	Thoroughbred	NA	Osteochondral injury and control	Serum and metacarpophalangeal or carpal joint synovial fluid bone turnover peptide biomarkers	NA
Desjardin *et al*. (2014)	QE	Foals in experimental station, France, NA	37 (Animal for each 3, tissues = 15)	Both 10-month	Anglo-Arabian foals	Case (M) Control(F)	Experimental mechanical loading on osteochondrosis affected samples or healthy samples	Cartilage and subchondral bone miRNA profiling	NA
Farries *et al*. (2019)	QE	Horses in training yard, Ireland, 2011-2012	Skeletal muscle 111(77/92, 60 at both time points)	Case (after training, 611.7 days) Control (757.5 days)	Thoroughbred	(64/47)	Before/after training	Transcriptome and genetic association (expression quantitative trait loci)	NA
Frisbie *et al*. (2010)	C	Three equine practice patients, USA, 2001	130 (59, 71)	2-3	Thoroughbred	51/79	Musculoskeletal injury or without injury	Serum bone turnover peptide biomarkers	# not having completed >2 months in the study, or sustaining multiple musculoskeletal lesions

Kim *et al.* (2018)	QE	Horse in race association. Korea, NA	4	11 (7–15)	Warmblood	Case and control (M, geldings)	Before/after exercise	Blood miRNA and mRNA profiling	NA
Kuemmerle *et al.* (2016)	CS	Horses from post-mortem Switzerland, NA	6	7.3 ± 4.6	Warmblood (5) Lipizzaner (1)	(3/3)	Free from any previous history of orthopaedic disease	mRNA profiling (transcriptome)	NA
Jackson *et al.* (2009)	C	Horses in 19 training yards, UK, NA	Two-year-old 529(60/469) Three-year-old 326(30/296)	2<br>3	Thoroughbreds	Two-year-old (231/298) Three-year-old (114/211)	Increased risk of fracture of 2- and 3-year-olds racehorses in the subsequent flat racing season	Serum bone turnover peptide biomarkers	#Fracture as a direct result of trauma (e.g. kick or fall)
Jackson *et al.* (2015)	C	Horses in training yards, UK, NA	100 (50/50) Three-year-old study 326(30/296)	2–3	Thoroughbred	NA	Carpal or fetlock joint injury or no injury	Serum bone turnover peptide biomarkers	NA

Lecchi *et al*. (2018)	CS	Equine practice patients, Italy, 2012 - 2014	18(9/9)	Case (9.4 ± 5.0) Control (2.4 ± 1)	Various breeds	Case (M) Control (mixed)	Acute laminitis with post treatment no prior treatment	Expression of targeted miRNA	NA
Mach *et al*. (2016)	QE	Horses in racing competition, France, NA	61	10 ± 2	pure-breed or half-breed Arabian	Case and control (20/41)	Before/after competing in three 160 km endurance competitions	Serum mRNA profiling	#8 horses due to poor metabolic condition or lameness
Mach *et al*. (2017)	QE	Horses in three different French competitions, France, NA	41	9.7 ± 1.5	pure-breed or half-breed Arabian	Case and control (28/13)	Before/after competing in three 160 km endurance competitions	Combined metabolome, transcriptome, and miRNome	#10 horses due to lameness, metabolic disorders
McGiney *et al*. (2017)	QE	Horses in training yards, Ireland, NA	31(Ci-miRNA = 20, skeletal muscle = 10)	Ci-miRNA (2) skeletal muscle (3)	Thoroughbre d	Ci-miRNAs (10/10) skeletal muscle (7/ 4)	Before/after exercise	Plasma miRNA profiling	#miRNAs with 80% missing data removed
Stefaniuk -Szmuki er *et al*. (2018)	QE	Horses in training yards, Poland, NA	10 (4/6)	2.5	Arabian	NA	Before/after exercise	Blood mRNA profiling	NA

Stefaniuk-Szmukier *et al*. (2019)	QE	Horses in training yards/racing, Poland, NA	53 blood from 23 horses	2.5	Arabian	NA	Before/after exercise/racing	Blood targeted mRNA	NA
Turlo *et al*. (2019)	C	Horses in training yards/racing, Poland, NA	Polish group 26(8/18) American group 35(6/29)	2	Thoroughbred	Polish group (11/15) American Group (17/18)	Musculoskeletal injury in during longitudinal training	Serum bone turnover peptide biomarkers	NA
Watts *et al*. (2017)	RCT	Horses recruited in University hospital, USA, 2013	17 (9/8)	Both 2 to 6	Quarter-Horse-type	Case (5/4) Control (2/6)	Collagenase-induced lesions with miR29a mimic and placebo-treated group	Serum Targeted miRNA	NA
Zavodovskaya *et al*. (2018)	CS	Horses in University hospital patients, USA,	16(8/8)	Case (20, 12 to 30) Control (17.5, 8 to 26)	Various breeds	Case (3/5) Control (4/4)	Silicate associated osteoporosis, Control	Bone marrow and tracheobronchial lymph node transcriptome	NA

Table S12. Study and participant characteristics for publications included in a systematic review of 19 human studies. RCT; Randomised controlled trial, QE; Quasi-Experimental Studies, CS; Cross-sectional, CC; Case-control, C; Cohort, F; female, M ; male, NA; not applicable, P-MP (postmenopausal), Pre-MP (pretmenopausal), OP (osteoporosis), VF (vertebral fracture), BMD (bone mineral density).

**Author (Year)**	**Study design**	**Study population**	**Sample size**	**Age**	**Gender (F/M)**	**Intervention**	**Outcome**	**Exclusion criteria (general reason for exclusion) # specific reasons for exclusion in each study**
Chatzipapas *et al.* (2009)	CC	Male military personnel, Greece, NA	64(32/32)	Case 22.91±2.99Control 22.91±3.21	All M	Stress fracture or healthy	Genetic association (SNPs)	NA
Feichtinger *et al.* (2018)	CS	University hospital patients, Austria,	Pre-MP = 10 P-MP = 10 Male with fracture = 16	Pre-MP (39.0 ± 8.6) Male (43.7 ± 11.1) P-MP (59.0 ± 11.4)	Pre-MP, P-MP F Male–M	Postmenopausal (P-MP) osteoporosis (OP), male idiopathic with low traumatic fractures or control Premenopausal (Pre-MP)	Serum targeted miRNA	#Secondary causes OP (e.g. diabetes mellitus type 1)
Feurer *et al.* (2019)	CS	Patients from health care insurer, Frace, 1992 - 1993	583<br>99	68.2<br>48.7	All F	P-MP women with fracture Pre-MP woman with fracture	Serum targeted miRNA	#OP-related drugs renal failure
Friedman *et al.* (2014)	CC	Israeli Defense Forces combat soldiers, Israel 2005 - 2010	DNA 94(34/60) from 308(161/14) soldiers	Case 20.1 ± 0.9 Control 20.1 ± 1.1	All M	Stress fractures by bone scan or without stress fractures	Genetic association (varitants)	NA
Hakansson *et al.* (2018)	QE	Elite endurance cyclists and peripheral artery disease patients from University hospital, Netherland, 2013	Exhaustive endurance exercise (EEE) =8 Time Trial 60 (TT) = 13 peripheral artery disease (PAD) = 16	EEE (22.9±1.2)TT60 (27.7 ±1.2) PAD (69 ± 13)	All M for EEE, TT60 PAD (87.5% male)	Before/after EEE, TT60, or cohort PAD	Serum small RNA profiling	#Confirmed or suspected malignancy and inability to give informed consent

Horak *et al.*(2018)	QE	Young male athletes from Sports Studies of Masaryk University, Czech Republic, NA	30	22.5 ± 4.06	All M	Before/after explosive 3 exercises week 5 and 8	Plasma targeted miRNA	NA
Kocijan *et al.* (2016)	CS	Patients in University hospital, Austria, NA	Case = 36 Control = 39	Case (46.6 ± 13.0) Control (46.6 ±9.4)	All F	Prevalent low-traumatic fractures or control	Serum miRNA profiling	Secondary OP or genetic disorders
Korvala *et al.* (2010)	CC	Patients from Defence Forces' military hospital, Finland, 2002 - 2003	192(72/120)	Case 20.3 ± 1.6 Control 18.9 ± 0.5	All M	Femoral neck stress fractures without stress fractures	Genetic association (SNPs)	NA
Ladang *et al.* (2019)	C	Patients recruited from a long-term prospective study (SarcoPhAge study), Belgium, NA	17<br>16	72.5 ± 5.2<br>73.8 ± 6.0	16/1<br>14/2	Fracture experience within 3 years without fracture	Blood targeted miRNA	#renal failure, 2 control subjects for analytical reasons
Mandourah *et al.* (2018)	CS	Patients in University hospital, UK, NA	161 (131/30)	Case (65.6 ± 9.5 67 ± 9.5 68.6 ± 10 70 ± 10) Control (67 ± 9.6)	(131/30)	Osteopaenia with/without fractures OP or healthy volunteers	Serum/plasma miRNA profiling	#under 18 years old, suffering from a disease unrelated to OP
Panach *et al.* (2015)	CC	Patients in Spain, NA	8/5	63.4 ± 8.1<br><br>79.6 ± 3.1	All F	Osteoporotic subcapital hip fracture or with severe osteoarthritis of the hip (controls)	Serum miRNA profiling	#Fractures due to high-energy trauma, any medication known to alter bone metabolism
Sansoni *et al.* (2018)	RCT	Healthy, physically active male adults, Italy, NA	9	24.3 ± 3.7	All M	Repeated sprints training or control	Blood targeted miRNA	other disease
Seeliger *et al.* (2014)	CS	Patients in University hospital, Germany, NA	Case = 40 Control = 40	Case 79.3 (66-89) Control 78.8 (67-91)	(77/3)	Femoral neck or pertrochanteric fracture or non-	Serum miRNA profiling	#Malignancy/benign ovarian cysts, Inflammation, chronic, systemic,

						osteoporotic control		metabolic disease
Sun *et al.* (2020)	CC	Patients in University hospital, China, NA	OP with vertebral fracture (VF) 6 OP without VF 6 control 6)	69.7(57–80) 68.0(43–80) 47.8(36–59)	6/0 5/1 4/2	OP with or without vertebral fracture, or control candidates	Blood miRNA profiling	#Any bone turnover treatment, tumours, chronic, metabolic diseases
Varley *et al.* (2018)	CC	Elite athletes From stress Fracture Elite Athlete (SFEA) cohort, Australia, NA	518(125/376)	Case (27.7 ± 7.5) Control (24.4 ± 5.4)	(69/449)	Stress fracture or without SF	Genetic association (SNPs)	NA
Weilner *et al.* (2015)	CS	Patients in University hospital Austria, NA	7 Validation 12 7 Validation 11	72.4 ±3.2 77.8 ±1.4 71.0 ±2.3 81.5 ±1.5	All F	P-MP F with fractures at the femoral neck P-MP F without fractures	Serum targeted miRNA	#Chronic treatment to affect bone metabolism
Yanovich *et al.* (2012)	CC	Israeli young active duty soldiers, Israel, 2007-2009	385(182/203)	Case 20.1 ± 1.7 Control 20.2±1.3	182 (17/165) 203 41/162	Stress fractures or without fractures	Genetic association (SNPs)	#Grade 1 or a single grade 2, metatarsal stress fracture
Yavropoulou *et al.* (2016)	CS	Patients in University hospital, Greece, NA	OP without VF 35 OP with VF 35 Control 30	68 ± 7 71 ± 7 68 ± 5	All F	P-MP women with osteopenia/ OP (the lumbar spine and/or total hip or femoral neck) With VF or Without VF	Serum targeted miRNA	#History of bone disease other than primary OP medication known to affect bone metabolism
Zarecki *et al.* (2020)	CS	Patients in University hospital, Austria, NA	35 24 17 40	67.9 ± 5.4 69.6 ± 7.0 69.6 ± 6.4 68.8 ± 6.5	All F	P-MP women without VF/low BMD with VF/low BMD without a treatment with VF/low BMD OP treatment healthy controls	Serum targeted miRNA	# Quality check, haemolysis, the lack of clinical information

Table S13. Quality appraisal and risk of bias was carried out using the JBI critical appraisal tool for randomised controlled trials, Quasi-Experimental Studies, Cross Sectional Studies, Case Control studies and cohort studies.

**Publications**	**C1**	**C2**	**C3**	**C4**	**C5**	**C6**	**C7**	**C8**	**C9**	**C10**	**C11**	**C12**	**C13**	**Number of Yes/total (Include/exclude)**
Randomised controlled trial/Experimental studies														
Sansoni *et al.* (2018)	Y	N	Y	N	NA	N	Y	Y	Y	Y	Y	Y	Y	9/13(Include)
Watts *et al.* (2017)	Y	Y	Y	NA	Y	Y	Y	Y	Y	Y	Y	Y	Y	12/13(Include)
Quasi-Experimental Studies (uncontrolled before and after studies, time series designs)														
Cappelli *et al.* (2018)	Y	Y	Y	Y	Y	Y	Y	Y	Y					9/9(Include)
Desjardin *et al.* (2014)	Y	Y	Y	Y	Y	Y	Y	Y	Y					9/9(Include)
Farries *et al.* (2019)	Y	Y	Y	Y	Y	Y	Y	Y	Y					9/9(Include)
Hakansson *et al.* (2018)	Y	Y	Y	N	Y	Y	Y	Y	Y					8/9(Include)
Horak *et al.* (2018)	Y	Y	Y	Y	Y	Y	Y	Y	Y					9/9(Include)
Kim *et al.* (2018)	Y	Y	Y	N	Y	Y	Y	Y	Y					8/9(Include)
Mach *et al.* (2016)	Y	Y	Y	N	Y	Y	Y	Y	Y					8/9(Include)
Mach *et al.* (2017)	Y	Y	Y	N	Y	Y	Y	Y	Y					8/9(Include)
McGivney *et al.* (2017)	Y	Y	Y	Y	Y	Y	Y	Y	Y					9/9(Include)
Stefaniuk-Szmuki er *et al.* (2018)	Y	Y	Y	N	Y	Y	Y	Y	Y					8/9(Include)
Stefaniuk-Szmuki er *et al.* (2019)	Y	Y	Y	N	Y	Y	Y	Y	Y					8/9(Include)
Cross sectional study														
Arai *et al.* (2008)	Y	Y	Y	Y	Y	Y	Y	Y						8/8 (Include)
Feichtinger *et al.* (2018)	Y	Y	Y	Y	Y	Y	Y	Y						8/8(Include)
Feurer *et al.* (2019)	Y	Y	Y	Y	Y	N	Y	Y						7/8 (Include)
Kocijan *et al.* (2016)	Y	Y	Y	Y	Y	Y	Y	Y						8/8(Include)
Kuemmerle *et al.* (2016)	Y	Y	Y	NA	NA	NA	Y	Y						5/8 (Include)
Mandourah *et al.* (2018)	Y	Y	Y	Y	Y	Y	Y	Y						8/8 (Include)
Seeliger *et al.*	Y	Y	Y	Y	Y	Y	Y	Y						8/8(Include)

(2014)												
Weilner *et al.* (2015)	Y	Y	Y	Y	Y	Y	Y	Y				8/8 (Include)
Yavropoulou *et al.* (2016)	Y	Y	Y	Y	Y	Y	Y	Y				8/8 (Include)
Zarecki *et al.* (2020)	Y	Y	Y	Y	N	N	Y	Y				6/8 (Include)
Zavodovskaya *et al.* (2018)	Y	Y	Y	Y	Y	Y	Y	Y				8/8(Include)
Case Control study												
Arens *et al.* (2013)	Y	Y	Y	Y	Y	N	N	Y	Y	Y		8/10(Include)
Blott *et al.* (2014)	Y	Y	Y	Y	Y	Y	Y	Y	NA	Y		9/10(Include)
Chatzipapas *et al.* (2009)	Y	Y	Y	Y	Y	Y	N	Y	Y	Y		9/10(Include)
Cleary *et al.* (2009)	Y	Y	Y	N	Y	N	N	Y	Y	Y		7/10(Include)
Friedman *et al.* (2014)	Y	Y	Y	Y	Y	Y	Y	Y	NA	Y		9/10(Include)
Korvala *et al.* (2010)	Y	Y	N	Y	Y	Y	Y	Y	Y	Y		9/10(Include)
Lecchi *et al.* (2018)	Y	Y	Y	Y	Y	Y	Y	Y	NA	Y		9/10(Include)
Panach *et al.* (2015)	Y	Y	Y	Y	Y	Y	Y	Y	N	Y		9/10(Include)
Sun *et al.* (2020)	Y	Y	Y	Y	Y	Y	Y	Y	N	Y		9/10(Include)
Varley *et al.* (2018)	Y	Y	Y	Y	Y	Y	Y	Y	NA	Y		9/10(Include)
Yanovich *et al.* (2012)	Y	Y	Y	Y	Y	Y	N	Y	N	Y		8/10(Include)
Cohort study												
Frisbie *et al.* (2010)	Y	Y	Y	Y	Y	Y	Y	Y	Y	Y	Y	11/11(Include)
Jackson *et al.* (2009)	Y	N	Y	Y	Y	Y	Y	Y	Y	Y	Y	10/11(Include)
Jackson *et al.* (2015)	Y	N	Y	Y	Y	N	Y	Y	Y	Y	Y	9/11(Include)
Ladang *et al.* (2019)	Y	Y	Y	Y	Y	Y	N	Y	Y	Y	Y	10/11(Include)
Turlo *et al.* (2019)	Y	N	Y	Y	Y	N	Y	Y	Y	Y	Y	9/11(Include)

JBI Critical Appraisal Checklist for Randomized Controlled Trials (RCT).

Criteria	Question	Yes	No	Unclear	NA
C1	1. Was true randomization used for assignment of participants to treatment groups?				
C2	2. Was allocation to treatment groups concealed?				
C3	3. Were treatment groups similar at the baseline?				
C4	4. Were participants blind to treatment assignment?				
C5	5. Were those delivering treatment blind to treatment assignment?				
C6	6. Were outcomes assessors blind to treatment assignment?				
C7	7. Were treatment groups treated identically other than the intervention of interest?				
C8	8. Was follow up complete and if not, were differences between groups in terms of their follow up adequately described and analyzed?				
C9	9. Were participants analyzed in the groups to which they were randomized?				
C10	10. Were outcomes measured in the same way for treatment groups?				
C11	11. Were outcomes measured in a reliable way?				
C12	12. Was appropriate statistical analysis used?				
C13	13. Was the trial design appropriate, and any deviations from the standard RCT design (individual randomization, parallel groups) accounted for in the conduct and analysis of the trial?				

Overall appraisal: Include Exclude Seek further info

JBI Critical Appraisal Checklist for Quasi-Experimental Studies (uncontrolled before and after studies, time series designs).

Criteria.	Question	Yes	No	Unclear	NA
C1	1. Is it clear in the study what is the 'cause' and what is the 'effect' (i.e. there is no confusion about which variable comes first)?				
C2	2. Were the participants included in any comparisons similar?				
C3	3. Were the participants included in any comparisons receiving similar treatment/care, other than the exposure or intervention of interest?				
C4	4. Was there a control group?				
C5	5. Were there multiple measurements of the outcome both pre and post the intervention/exposure?				
C6	6. Was follow up complete and if not, were differences between groups in terms of their follow up adequately described and analyzed?				
C7	7. Were the outcomes of participants included in any comparisons measured in the same way?				
C8	8. Were outcomes measured in a reliable way?				
C9	9. Was appropriate statistical analysis used?				

Overall appraisal: Include Exclude Seek further info

JBI Critical Appraisal Checklist for Cross Sectional Studies (CS).

Criteria	Question	Yes	No	Unclear	NA
C1	1. Were the criteria for inclusion in the sample clearly defined?				
C2	2. Were the study subjects and the setting described in detail?				
C3	3. Was the exposure measured in a valid and reliable way?				
C4	4. Were objective, standard criteria used for measurement of the condition?				
C5	5. Were confounding factors identified?				
C6	6. Were strategies to deal with confounding factors stated?				
C7	7. Were the outcomes measured in a valid and reliable way?				
C8	8. Was appropriate statistical analysis used?				

Overall appraisal: Include Exclude Seek further info

JBI Critical Appraisal Checklist for Case Control study (CC).

Criteria	Question	Yes	No	Unclear	NA
C1	1. Were the groups comparable other than the presence of disease in cases or the absence of disease in controls?				
C2	2. Were cases and controls matched appropriately?				
C3	3. Were the same criteria used for identification of cases and controls?				
C4	4. Was exposure measured in a standard, valid and reliable way?				
C5	5. Was exposure measured in the same way for cases and controls?				
C6	6. Were confounding factors identified?				
C7	7. Were strategies to deal with confounding factors stated?				
C8	8. Were outcomes assessed in a standard, valid and reliable way for cases and controls?				
C9	9. Was the exposure period of interest long enough to be meaningful?				
C10	10. Was appropriate statistical analysis used?				

Overall appraisal: Include Exclude Seek further info

JBI Critical Appraisal Checklist for Cohort Studies.

Criteria	Question	Yes	No	Unclear	NA
C1	1. Were the two groups similar and recruited from the same population?				
C2	2. Were the exposures measured similarly to assign people (horses) to both exposed and unexposed groups?				
C3	3. Was the exposure measured in a valid and reliable way?				
C4	4. Were confounding factors identified?				
C5	5. Were strategies to deal with confounding factors stated?				
C6	6. Were the groups/participants free of the outcome at the start of the study (or at the moment of exposure)?				
C7	7. Were the outcomes measured in a valid and reliable way?				
C8	8. Was the follow up time reported and sufficient to be long enough for outcomes to occur?				
C9	9. Was follow up complete, and if not, were the reasons to loss to follow up described and explored?				
C10	10. Were strategies to address incomplete follow up utilized?				
C11	11. Was appropriate statistical analysis used?				

Overall appraisal: Include Exclude Seek further info

Table S14. Summary of results for primary and secondary outcomes including assessment of evidence. CTX-I (carboxy-terminal telopeptide fragments of type I), BAP (bone-specific alkaline phosphatase), COMP (oligomeric matrix protein), CTX-II (carboxy-terminal telopeptide fragments of type II), CS846 (aggrecan chondroitin sulfate 846 epitope), CPII (type II collagen synthesis), GAGs (glycosaminoglycan), Col CEQ (type II collagen degradation), OC (osteocalcin), PICP (carboxy-terminal propeptide of type I collagen), ICTP (crosslinked carboxy-terminal telopeptide of type I collagen), ROC (receiver operating characteristic), VDR (vitamin D receptor), CTSK (cathepsin K).

Study	Samples/Analytic methods/ Normalisation	miRNA	miRNA target or mRNA	Validation/findings	Assessment of evidence
**miRNAs/protein analysis/ gene expression related to stress fractures or biomarkers for musculoskeletal injuries in horses**					
Arens et al. (2013)	48 horses; 20 scapula stress fracture cases and 28 controls. **Targeted biomarker candidate study in horse** CTX-1 and BAP activity	NA	Serum biomarkers were not accurate for bone fragility syndrome diagnosis	No independent cohort validation None of the tests evaluated were accurate enough to replace scintigraphy for mild disease; physical examination and scapular ultrasonography accurate at moderate to severe bone fragility syndrome	Low
Arai *et al*. (2008)	51 samples from 26 osteophyte + and 25 osteophyte (–) with carpal bone fracture synovial fluid (SF), serum and urine and the development of osteochondral damage and osteophyte formation **Targeted biomarker candidate study in horse** ELISA to COMP	NA	The COMP measurements obtained using the two monoclonal antibodies were highly correlated between SF, serum, or urine. Horses with osteophyte (+) showed ↑urinary COMP (μg)]/[urinary creatinine (mg)] ratio	No independent cohort validation Urinary level of COMP is more dependent on osteoarthritis pathology following carpal fracture than the serum level. Classification as osteophyte formation in early osteoarthritis and fracture in severe osteoarthritis sound be questioned	Low
Blott *et al*. (2014)	Catastrophic distal limb fractures (n = 269) and . Control horses (n = 253)	NA	Significant genetic variation associated with fracture	No independent cohort validation Genetic variance explained by SNPs for fracture risk was	Low

	**Non-biased genetic association study** DNA sample from bone marrow biopsies (cases) or blood samples (controls) Genotyping using SNP50 BeadChip (54,602 SNP assays)		risk on chromosomes 9, 18, 22 and 31. Three SNPs on chromosome 18 and one SNP on chromosome 1 reached genome-wide significance (p < 0.05). Two of the SNPs on ECA 18 located in a haplotype block containing the gene zinc finger protein 804A (ZNF804A). One haplotype within this block (1.95 times at less risk of fracture than cases); a protective effect, while a second haplotype increases fracture risk (cases at 3.39 times higher).	estimated to be 0.479. Genetic variance estimates for each individual chromosome showed significant variance on chromosomes 9, 18, 22 and 31	
Cleary et al. (2010)	Serum and metacarpophalangeal or carpal joint synovial fluids (SF) with (n=38 radiographs and	NA	CTX-II in SF, CTX-II in SF/ CTX-II in serum ratio ↑ with joint injuries	Independent cohort validation (14 samples) serum and SF CTX-II concentrations and SF:serum CTX-II ratio, 64% to 93% of serum and SF samples were	Low

	arthroscopy) /without (n=40) osteochondral injury **Targeted biomarker candidate study** CTX-II concentrations determined by an ELISA		compared with pre- and post-exercise Serum CTX-II in post-exercise and injured-horse ↓ than pre-exercise samples arthroscopic scores were not correlated with SF or serum CTX-II concentration	correctly classified into their appropriate group (pre-exercise, postexercise, or injured-joint samples)	
Desjardin et al. (2014)	epiphyseal cartilage and subchondral bone from10 months Anglo-Ararb foals from the same stallion with healthy (n=3) or radiographically determined tarsal osteochondrosis (n=3) **Non-biased miRNAs profiling study** Small RNA-seq Mechanical loading (compression) quantitative PCR /miR-125a-5p, miR-331	1. Osteochondrosis Cartilage; 49 DE miRNAs (3 ↑, 46 ↓ miR-126-5p, 135a-5p, 451, 486-3p, 486-5p ↓) Bone; 41 DE (1 ↓, 40 ↑ miR-1249, 18a-3p, 197, 296-5p, 423-3p, 486-3p, 486-5p, 92a-3p ↑) 2. Mechanical loading Cartilage; 19 DE miRNAs (12 ↑, 7 ↓ miR-17-3p ↑, 874-3p ↓) Bone; 21 DE miRNAs (9 ↑, 12 ↓ miR-206-3p, 17-3p).	About 2400 putative targets identified in cartilage, cell cycle and differentiation, energy production and metabolism as well as extracellular matrix structure. In bone, osteoblasts and osteoclasts differentiation, regulation of energy production, vesicle transport and growth factor signalling pathways	No independent cohort validation but validated by qPCR in tissues quantitative PCR osteochondrosis compared to heathy control Cartilage 9 miRNAs were downregulated miR-126-5p, miR-486 3p ↓ Bone 9 miRNAs were upregulated, miR-1249, miR-197 ↑	Low

		3. Osteochondrosis loaded compared healthy loaded<br>Cartilage; 15 DE miRNAs (8 ↑, 7 ↓ miR-874-3p ↑)<br>Bone; 28 DE miRNAs (24 ↑ 4↓, miR-1-3p ↑, miR-2887↓)			
Frisbie *et al*. (2010)	Blood (serum/plasma) from 2-3-year-old racehorses with injury (n=59) or without injury (n=71)<br>**Targeted biomarker candidate study in horse**<br>CS846, CPII, GAGs, Col CEQ, CTX, type I and II collagens (C1, 2C), OC<br>ELISA, enzyme immunoassay	NA	No significant differences between injury and control at the baseline or entry time point (a trend for the intra-articular fragmentation horses lower CPII)<br>Monthly longitudinal samples, CS846 and GAGs levels ↓ for the intra-articular fragmentation and dorsal metacarpal disease groups compared to control, low CPII for dorsal metacarpal disease, OC ↓ longitudinally throughout the	No independent cohort validation<br>The greatest changes occur 4–6 month prior to injury (longitudinal sampling is critical)<br>4 biomarkers around 6 and 2 months prior to injury, ↓ articular cartilage biomarkers (GAGs, CS486) and an ↑ bone biomarker (CTX, OC)<br>↑ GAG or ↓ OC with exercise, a normal adaptive response<br>↓ CS846 6 months prior to injury or until after injury may suggest an impaired synthetic response	Low

			study, CS846 ↓ in intra-articular fragmentation 6 months prior to injury<br>CTX ↑ at 7 and one months prior to injury and at one month preinjury than the post injury sample. Similarly, OC ↑ at 6 months preinjury and between 7 and 4 months preinjury		
Jackson *et al.* (2009)	Blood samples collected from 529 two-year-old of which 60 developed a fracture and 326 three-year-old of which 30 fractured. Cases followed for development of fracture using radiography and nuclear scintigraphy<br>Serum **targeted biomarker candidate study**<br>OC, PICP, ICTP and CTX-I measured by ELISA	NA	No significant differences were identified between fracture and non-fracture groups for any serum biomarker	No independent cohort validation<br>NA	Moderate
Jackson *et al.*	50 TB racehorses sampled post injury to	NA	fetlock joint injuries showed	No independent cohort validation<br>ROC curve analysis	Low

(2015)	the carpal or fetlock joint (no advanced imaging and imaging and diagnostic analgesia not always used) and 50 controls. Serum **targeted biomarker candidate study** CPII, CS846, 2 cartilage degradation biomarkers and a serum bone formation marker BAP by ELISA kits		↓ serum CPII and ↑ serum BAP activities compared to controls CTX-II ↓ following joint injury.	CPII concentration good sensitivity (82%), low specificity (50%) Despite statistical significance being reached not enough difference to use as clinical biomarkers for diagnosis or prognosis	
Kuemmerle *et al*. (2016)	Samples from 6 horses, tendon, collateral ligaments, cancellous bone from the distal metaphysis of the third metacarpal bone; cartilage from the articular surfaces of the MCP joint **Non-biased mRNAs tissue profiling study in horse** RNA-seq	NA	12 genes that were selectively expressed in either tendon (THBS4, TENM4, SCX, ENPEP), ligament (TNMD), bone (BTLN9, CD36, MASP2, SNCG), or cartilage (CHODL, ACAN, THBS3), and two genes that were exclusively expressed in tendon (EYA2 and GPRIN3) EYA2 and GPRIN3 as specific markers of equine tendon as	Independent cohort validation (3 horses) EYA2 expression levels were significantly increased in equine tenocyte microtissue spheroids as compared to GPRIN3 and the selective cartilage marker CHODL	Low

			compared to ligament, bone and cartilage.		
Lecchi et al. (2018)	9 acute laminitis and 8 control stallions blood sampled. Serum **targeted miRNA candidate study** quantitative PCR Behavioural recordings Composite Pain Scale (CPS) and horse grimace scale (HGS) Computational target prediction of miR targets /spike-in cel-miR-39	**miR-23b-3p, miR-145-5p** and **miR-200b-3p** ↑ in acute laminitis potential biomarkers of pain) ROC was then used to evaluate the diagnostic performance of miRNAs miR-200b-3p ↓ after routine laminitis treatment	target mRNA - ion channel families (SLC, KCN, SCN, GABA receptor, CACN, ADRA, KCTD, HCN, TPCN, TRP) and the glutamatergic pathway (GLNT, EAAT, VGCC) major excitatory neurotransmitter released in synapse of the pain transmitting afferent neurons	No independent cohort validation Combining two miRNAs, **miR-145-5p and miR-200b-3p**, increased efficiency in distinguishing animals with acute pain deregulated miRNAs were positively correlated to HGS scores.	Moderate
Turlo *et al.* (2019)	Prospective study 28 racehorses, 8 developed musculoskeletal injury and 18 did not. Serum **targeted biomarker candidate study** concentrations of bone and cartilage biomarkers OC, CTX I, GAG, CP II using ELISA kits	NA	Mean GAG and CPII levels ↓ in injured group compared to control ↓ OC and CTX-I levels in injured horses comparing to controls in polish population	Matching archival data used (35 samples) for logistic regression analysis Changes in serum GAG and CPII in racehorses at risk of injury appear to be similar across distinct populations while dynamics of serum bone marker is more population-specific	Low
Watts et al. (2017)	superficial digital flexor tendon collagenase	miR-29a↓ throughout a time course of induced	miR-29a mimic in equine tenocytes selectively reduced	No independent cohort validation but miR-29a target validated by qPCR Ultrasound; lower CSA in treated tendons	Moderate

	animal model, 9 treated and 8 controls **Targeted miRNA candidate study** miR-29a mimic treatment of explants from 3 normal horses euthanised unrelated to study quantitative PCR /RNU6-6P (U6B small nuclear RNA)	tendon injury (week 3,5, 9,17)	COL3A1 early in the model but not COL1A1 mRNA	MRI placebos showed increased T2 relaxation at 12 weeks	
Zavodovskaya *et al.* (2018)	Rib bone marrow, tracheobronchial lymph Nodes from 8 equine with silicate associated osteoporosis or 8 control horses **Non-biased mRNAs profiling study** RNA-seq	NA	Bone marrow; ↑ SLC9A2, OMD, DLX3, TRPV4, COL24A1, SP7, Osx, BSP, BGLAP, OC, SMPD3, COLXIA2, CHAD, ANO5, WFDC1, CDH15, DAPK2 ↓HSD17B6 Lymph nodes; ↑PTX3, FCAR, DMP1	No independent cohort validation hyperplastic and hypertrophic osteoblast in bone marrow from osteoporosis horses ↑ osteoblastic activity; pathological mechanism for osteoporosis or a compensatory of osteolysis	Low
**microRNAs in response to exercise and mechanical loading in horses and human**					
Cappelli *et al.* (2018)	4 endurance horses samples before exercise and within 2 hours of completing a 90km endurance competition Blood/plasma **Non-biased miRNAs profiling study in horse**	involved in exercise responses ↑miR-206, miR-208b, miR-133a miR-1, miR-133b, miR-499-5p, miR-95, miR-224, miR-381	12 miRNAs, the 6 most downregulated and the 6 most up-regulated miRNAs Muscle remodeling (IGF1R, EGFR, PURB, TAGLN,	No independent cohort validation	Low

	miRCURY ™ RNA Isolation Kit—Biofluids<br>Small RNA-seq	↓ miR-361-3p, miR-1180, miR-486-3p, miR-504, miR-328, miR-100, miR-296, miR-6529, miR-9177, miR-9021<br>miR-486-5p	TMOD2, LASP1, SGCD)<br>Energy metabolism and cellular homeostasis maintenance (various solute carriers, such as SLC5A3, SLC1A2, SLC7A1, STX6.<br>Inflammatory response and modulation of cell migration (IGF1R, EGFR, BCL2, PTMA)		
Farries et al. (2019)	muscle biopsies from 111 horses for RNA seq DNA from whole blood for genotyping at rest or after high-intensity exercise<br>**Non-biased mRNAs profiling study in horse**<br>RNA-Seq<br>**Genetic association study**<br>Genotyping	NA	4,992 *cis*- expression quantitative trait loci (eQTL) associated with the expression of 1,922 genes post-exercise; 1,703 *trans*-eQTL associated with 563 genes at rest; and 1,219 *trans*-eQTL associated with 425 genes post-exercise. The gene with the highest *cis*-eQTL association at both time-points was the endosome-	No independent cohort validation<br>Functional analysis of genes with significant eQTL revealed significant enrichment for cofactor metabolic processes. heritable variation in genomic elements such as regulatory sequences	Low

			associated-trafficking regulator 1 gene (*ENTR1)*, Post-exercise:, a potential role in the transcriptional regulation of the solute carrier family 2 member 1 glucose transporter protein (SLC2A1)		
Hakansson et al. (2018)	Blood from 8 elite cyclists and 16 patients with peripheral artery disease **Targeted miRNA candidate study in human** quantitative PCR SnoRNA expression was normalized to U6 miRNA-191 for miRNA	1)**miR-193a-5p, miR-29a-3p** ↑ response to exercise miR-99b-5p and miR-151a-3p ↓ in the recovery process 2) miR-133b-3p and possibly miR-1-3p were regulated in response to exercise after the 60 min time trial. 3) SNORD114.1 ↑in response to exercise	**miR-29a-3p** a crucial role in the formation and maintenance of the extracellular matrix, the production of collagens **miR-193a** interacts with High mobility group box-1 (HMGB1) on angio- and vasculogenesis miR-106b overexpression result in skeletal muscle mitochondrial dysfunction and insulin resistance	Independent cohort validation (8 elite cyclists) miR-29a-3p most consistently regulated miR-29a-3p and miR-193a-5p ↓in response to a 60 min time trial Critical miR-106b-5p recovery after exercise microRNAs miR-29a-3p and miR-193a-5p may help us distinguish between repeated exhaustive and non-exhaustive endurance exercise	Low
Horak *et al.*	30 young males recruited to 3 training	miR-21 and miR-93 levels ↑after 5 weeks	611 verified target genes	No independent cohort validation	Low

(2018)	programmes: explosive training, hypertrophic muscle training and high intensity interval training<br>Plasma **targeted miRNA candidate study in human** (miR-21, miR-222, miR-16 and miR-93 levels)	then ↓to below the initial level after 8 weeks of exercise, while miR-16 gradually ↓EXPL ↓ in miR-222 after 5 weeks of exercise, which then remained stable<br>HYP exercise ↑ in miR-93, miR-16 and miR-222 after 5 weeks of intervention and a subsequent decrease	Of these, further selected genes linked to exercise-induced physiological adaptations (i.e. muscle growth, mitochondrial synthesis, angiogenesis, etc)<br>miR-16 and -21 target VEGF and HIF-1α<br>miR-222 target cardiac growth and protect against ischaemic heart damage	miR-16, miR-21, miR-222 and miR-93 are significantly changed during explosive strength hypertrophic strength training and high-intensity interval training. miR-21 and miR-93 increased at the beginning of training but is not sustained. mi-R-16 reduced with training. miR-222 decreased in explosive strength group but not hypertrophic muscle group	
Kim *et al.* (2018)	4 Warmblood horses samples pre- and post-exercise blood<br>**non-biased miRNAs profiling study in horse**<br>NGS analysis (small RNA-seq) for miRNA<br>mRNA profiling (microarray)	Exercise-induced DEs<br>Including ↑**miR-423-5p, ↓ miR-144, miR-33a, miR-545.** Novel miR-14-5p present before exercise but was not detected after exercise. Novel miR-95-3p was not detected pre-exercise but was detected after exercise	targeted 1,625 genes and 905 genes<br>miR-144 targeted 335 genes, miR-33a targeted 353 genes, miR-423-5p targeted 367 genes, miR-545 targeted 570 genes, novel miR-14-5p targeted 115 genes, and novel miR-95-3p targeted 790 genes.	No independent cohort validation<br>The networks of five mRNAs (LOC100050849, LOC100054517, KHDRBS3, LOC100053996, and LOC100062720) matched to the predicted target genes of the 6 miRNAs<br>Small sample size	Low

Mach *et al.* (2016)	14 French endurance horses in experimental group and 47 horses in the validation group sampled before and after exercise<br>Serum for biochemical assays<br>Whole blood for transcriptome and miRNome profiling<br>**Non-biased miRNAs profiling study in horse**<br>microarray for miRNome	167 DE miRNAs including 12 equine-specific DEmiRNAs and 19 putative novel miRNAs<br>91 DEmiRNAs (↑70↓ 21) an experimentally annotated targetome, miR-138-5p and miR-26b-5p significantly more predicted targets<br>miR-15a, miR-16, miR-17, miR-18a, miR-20a,b, miR-21-5p, miR-27a, miR-30b, miR-93, miR-101, miR-106, miR-107, miR-125b, miR-130, miR-138-5p, miR-145, miR-181b-5p, miR-221, miR-223, miR-342-3p and miR-505 previously reports on exhaustive exercise in humans	2,453 DEGs by mRNA microarray<br>↑1,165 associated with the inflammatory response, intestinal permeability and regulation of the response to stress and bacterium<br>↓1,288 mRNAs related to macromolecule, catabolism, cellular respiration, mitochondrial transport, and transcriptional and translational activity<br>The main putative regulatory TF; ZFP42, followed by SPI1, FOXO3, IRF3 and NRF1, the EP300 protein stimulated transcription of miR-92a, which in turn suppressed EP300 expression	Independent cohort validation using graphical Gaussian models (47 horses)<br>Validated by qPCR<br>miR-181b-5p and miR-505-5p, miR-21-5p; suggesting that these miRNAs might be involved in the regulation of exercise-related physiological processes	Low
Mach *et al.*	10 French endurance horses in experimental	5 miRNAs whose expression was	7,678 DEGs	Independent cohort validation (31 horses) for multiple factor analysis	Low

(2017)	group and 31 horses in the validation group sampled pre- and post-exercise Blood **non-biased miRNAs profiling study in horse** proton nuclear magnetic resonance (1H NMR)-based metabolomic analysis microarrays	significantly altered after exercise 107 DEmiRNAs (let-7b-5p, miR-16-5p, miR-21-5p, miR-92a-3p, and miR-192-5p regulated more than ten metabolic genes each)	network based on 11 unique metabolites, 263 metabolic genes post-competition characterized by lactate, glycerol, creatine, urea, and aromatic amino acids including various metabolism Foxo and glucagon signaling pathways to induce gluconeogenesis, and the relationship between lactate and metabolic genes encoding proteins	strong associations between lactate, methylene derivatives, miR-21-5p, miR-16-5p, let-7 family and genes that coded proteins involved in metabolic reactions primarily related to energy, ubiquitin proteasome and lipopolysaccharide immune responses	
McGivney *et al.* (2017)	20 three-year TB horses in sprint training and 11 two- year TB horses samples pre and post exercise. Blood (ci-miRNA) Muscle (gluteus medius biopsies) **non-biased miRNAs profiling study in horse** 7/20 samples failed quality control	Haemolysis levels were greater than the threshold therefore no exercise-induced changes in plasma ci-miRNA could be evaluated Skeletal muscle miRNA, 5 DE miRNAs (miR-21-5p, let-7d-3p, let-7d-5p, miR-30b-5p,	1077 of the predicted targets to these 2 miRNAs while only 3 gene targets were predicted for let-7d-3p. the Jak-STAT, MAPK, insulin and mTOR signaling pathways	No independent cohort validation Haemolysis above the accurate detection threshold occurred in 18/25 resting samples and all post-exercise samples Human panel may have limited detected of equine miRNAs	Low

	TaqMan Array microRNA Assays/ Exogenous RNA spike-ins (UniSp2, UniSp4, UniSp5, cel-miR-39-3p)	miR-30e-5p) between time-points.			
Sansoni et al. (2018)	Blood samples collected at start, weeks 4 and 8 after sprint training of the experimental group (n=9) and a control group without sprint training (n=9). Serum **targeted miRNA candidate study in human** quantitative PCR (miR-21-5p, miR-23a-3p, miR-24-3p, miR-93-5p, miR-100-5p, miR-122-5p, miR-124-3p, miR-125b-5p, miR-148a-3p, miR-637) /miR-425-5p and miR-484 as reference	miR-21-5p stable 8-week repeated-sprint training changed expression of circulating miRNA (miR-23a-3p, miR-24-3p, miR-100, miR-122-5p, miR-125-5p, and miR-148a-3p ↓, while miR-93-5p ↑).	Serum DKK1, sclerostin (SOST), osteoprotegerin, OC, Osteopontin, cytokines (IL-1β, TNFα), and metabolic hormones (leptin, insulin, PTH) None of the cytokines or metabolic hormones changed significantly while, the bone markers, DKK1, OC and sclerostin were slightly but significantly decreased	No independent cohort validation 8-week repeated-sprint training downregulates the expression of circulating miRNA associated with fracture risk	Low
Stefaniuk *et al.* (2018)	6 two-year-old Polish Arab horses blood sampled before training and 4 different horses sampled after 24 weeks of racing training	NA	deregulated pathway involved in bone homeostasis (osteoclast differentiation)	Validation in the same cohort by quantitative PCR PDLIM7, TYROBP, TXLNG, TCAP, CLEC5A, BGLAP	Low

	**Non-biased mRNAs profiling study in horse** RNA-seq		Among the significantly expressed molecules, BGLAP, CTSK, TYROBP, PDLIM7, SLC9B2, TWSG1, NOTCH2, IL6ST, VAV3, NFATc1, CLEC5A, TXLNG.		
Stefaniuk *et al.* (2019)	23 Arab horses blood sampled repeatedly for 3-year-old race training and competing in flat races **Targeted mRNA candidate study in horse** osteoclastogenic related genes NFATc1, CTSK, DAP12, CLEC5A, IL6ST, VAV3		training induced changes in transcript abundance of genes (NFATc1, CTSK, DAP12, CLEC5A, IL6ST, VAV3) involved in osteoclastogenesis hence bone resorption	No independent cohort validation The initial training had greater effect on expression pattern than increased, prolonged, established conditioning	Low
**Genetic association studies related to stress fractures in young adults (e.g. athletes/military recruits) equivalent to equine stress fracture**					
Chatzipapas *et al.* (2009)	Blood from a cohort of Greek male military personnel beyond basic training including 32 patients with stress fractures (n=32) or healthy volunteers (n=32)	NA	The f allele FokI more frequent in patients, while the B allele showed such a tendency. a 2.7-fold and a 2.0-fold increase in risk of stress fractures	No independent cohort validation	Low

	**Targeted genetic association study** PCR-RFLP method FokI, BsmI, ApaI, and TaqI polymorphisms of the VDR gene		associated with the f and B alleles (OR, 2.7, 95% CI, 1.2–5.9; p=0.014 and OR, 2.0, 95% CI, 1.0–4.1; p=0.053). No statistically significant association at t or an alleles. ↓ Tscores associated with the presence of f and B alleles. Mean values of T-scores of SI ↓ in patients		
Friedman *et al.* (2014)	Blood DNA pool of stress fracture cases (n=34) and controls (n=60) from a cohort of Israeli Defense Forces **Non-biased genetic association study** Next generation sequencing	NA	146 candidate sequence variants for further individual genotyping NEB, GRK4, SLC6A18, LRRC55, SIGLEC12 and ELFN2 cell development, morphology, survival and death, cell-to-cell signalling and interaction, humoral immune response, inflammatory response, nervous	Independent cohort validation (136 cases and 127 controls) three missense variants (rs7426114, rs4073918, rs3752135 in the NEB, SLC6A18 and SIGLEC12 genes, respectively) and three synonymous mutations (rs2071856, rs2515941, rs716745 in the ELFN2, GRK4, LRRC55 genes)	Low

			system development and function, and tissue development		
Korvala *et al.* (2010)	Blood from 72 Finnish military conscripts with a femoral neck stress fracture or 120 controls **Targeted genetic association study** PCR, sequencing COL1A1, COL1A2, OPG, ESR1, VDR, CTR, LRP5, IL-6) interaction between the CTR (rs1801197) and the VDR C-A haplotype was observed	NA	lacking the C allele in CTR and/or the C-A haplotype in VDR - a 3-fold higher risk of stress fracture than subjects carrying both (OR = 3.22, 95% CI 1.38-7.49, p = 0.007). the LRP5 haplotype A-G-G-C alone and in combination with the VDR haplotype C-A was associated with stress fractures through reduced body weight and BMI	No independent cohort validation	Low
Varley *et al.* (2018)	Saliva DNA from British elite athletes with stress fractures (n = 125) or without stress fractures (n = 376) **Targeted genetic association study** 11 SNPs investigated proprietary fluorescence-based	NA	SOST SNP rs1877632 and VDR SNPs rs10735810 and rs731236 with stress fracture. In the whole cohort, SOST SNP rs1877632 heterozygotes and homozygotes of the	No independent cohort validation	Low

	competitive allele-specific PCR		rare allele combined made up 59% of stress fracture In the multiple stress fracture cohort, homozygotes of the rare allele of VDR SNPs rs10735810 and rs731236 association with stress fracture		
Yanovich *et al.* (2012)	Blood from a cohort of Israel active duty soldiers with fractures (n=182) or without fractures (n=203) **Targeted genetic association study** 268 SNPs analysis within 17 genes	NA	25 polymorphisms within 9 genes (NR3C1, ANKH, VDR, ROR2, CALCR, IL6, COL1A2, CBG, and LRP4) showed statistically significant differences 17 genetic variants (9 SNPs and 8 Haplotypes) were associated with an increased stress fracture risk, and 8 variants (7 SNP and 1 haplotype) with a decreased stress fracture risk.	No independent cohort validation None of the SNP associations remained significant after correcting for multiple comparisons (false discovery rate- FDR).	Low
**miRNAs related to osteoporotic fragility fracture in humans**					

Feichtinger et al. (2018)	36 idiopathic osteoporosis fractures, 10 pre and 10 postmenopausal women that had sustained fractures. Blood/serum Bone biopsy **Targeted miRNA candidate study** quantitative PCR	miR-29b-3p, miR-324-3p, and miR-550a-3p showed significant correlations to histomorphometric parameters of bone formation as well as microstructure parameters. miR-29b-3p and miR-324-3p ↓ in patients undergoing anti-resorptive therapy	NA	No independent cohort validation Correlations between miRNAs and dynamic bone parameters Four miRNAs were found to be correlated to bone microarchitecture and seven miRNAs to dynamic histomorphometry	Low
Feurer *et al.*(2019)	Serum from 99 premenopausal women (one woman with a prevalent fracture) or 583 postmenopausal women (122 women with fractures) **Targeted miRNA candidate study** normalizing with UniSp6	None of the miRNAs showed a significant difference by the menopausal status after adjustment for age. subgroups according to the duration of menopause to low BMD vs normal BMD (osteoporosis, non-osteoporosis, osteopenia) with fracture vs without fracture; no significant difference after adjustment for age	OC weakly associated with 21 of the 32 miRNAs other bone remodelling biomarkers (CTX, P1NP, and BAP) not associated with the serum levels of the miRNAs	No independent cohort validation Correlations between miRNAs and dynamic bone parameters No evidence that these 32 preselected miRNAs were not associated with BTMs, BMD, microarchitecture, and or fragility fractures	Low

Kocijan *et al*. (2016)	Serum from osteoporosis patients with prevalent low-traumatic fractures (n=36) or without fractures (control n=39) **Non-biased miRNA profiling study** 187 circulating miRNAs quantitative PCR panel	DE miRNAs were identified in all (46 in premenopausal, 52 in postmenopausal, 55 in male). 19 miRNAs common Eight miRNAs (miR-152-3p, miR-30e-5p, miR-140-5p, miR-324-3p, miR-19b-3p, miR-335-5p, miR-19a-3p, miR-550a-3p) excellent discriminators of patients with low traumatic fractures, regardless of age and sex.	Correlation analysis identified significant correlations between miR-29b-3p and P1NP, and miR-365-5p and iPTH, TRAP5b, P1NP and OC, as well as BMDL1–L4 and miR-19b-3p, miR-324-3p, miR-532-5p, and miR-93-5p.	No independent cohort validation Correlations between miRNAs, numerical clinical parameters and bone turn over biomarkers using laboratory analyse significant correlations between miR-29b-3p and P1NP, and miR-365-5p and iPTH, TRAP5b, P1NP and Osteocalcin, as well as BMDL1–L4 and miR-19b-3p, miR-324-3p, miR-532-5p, and miR-93-5p	Low
Ladang *et al*. (2019)	Serum from Belgian residents over 65-year-old who developed fractures within 3 years of the time of collecting the serum (n=17 persons) or who did not experience fractures in the same period (n=16)**Targeted miRNA candidate study**	none of the 19 other miRNAs showed a difference a trend of higher OsteomiR° scores in fracture subjects OsteomiR° score ↑ in low BMD		No independent cohort validation ROC curve analysis AUC= 0.687 Sensitivity 76%, specificity 63%, predictive positive value 68% negative predictive value 71% single miRNA is not sufficient to predict fragility fracture risk	Low

	quantitative PCR (OsteomiR test kit) normalized with Unisp4				
Mandourah *et al.* (2018)	76 osteoaenia, 51 osteoporosis and 12 controls over age 40 years. Blood **Non-biased miRNA profiling study in human** miRNA PCR arrays quantitative PCR	hsa-miR-122-5p and hsa-miR-4516 were statistically significantly differentially-expressed between non-osteoporotic controls, osteopaenia and osteoporosis patients. hsa-miR122-5p and hsa-miR4516 in clinical samples also independently showed a strong significant correlation with BMD lumbar spine T-score	Bioinformatic analysis of intracellular target mRNAs BMP2K, FSHB, IGF1R, PTHLH, RUNX2, SPARC, TSC22D3 and VDR, CYP3A4, MAPK1, CNR2, CYP17A1, CYP19A1, MAPK3 and CNR1	Independent cohort validation (139 serum samples and 134 plasma samples) Both microRNAs (hsa-miR-122-5p and hsa-miR-4516) were combined together, there was a much stronger diagnostic value for osteoporosis (AUC = 0.75, P = 0.004) than either separately	Low
Panach *et al.* (2015)	Serum from 15 women with osteoporotic subcapital hip fracture or 12 women with severe osteoarthritis of the hip that required the implantation of a hip prosthesis as controls	42 DE miRNAs ↑miR-144-5p, miR-21-5p, miR-101-3p, miR-210, miR-122-5p, miR-423-5p, and miR-155-5p in fracture group; ↑ miR-143-3p, miR-497-5p, miR-125b-	bone metabolism (miR-143-3p, miR-122-5p, miR-125b-5p, miR-210, and miR-21-5p) plus miR-34a-5p since this miRNA appears to be a novel and critical	Independent cohort validation (27 samples) ↑ miR-122-5p, miR-21-5p, miR-125b-5p value as biomarkers for distinguishing fracture patients from osteoarthritic controls miR-21-5p correlated to CTX	Low

	**Non-biased miRNA profiling study in human**<br>179 microRNA PCR Panel<br>normalized with miR-93-5p	5p, miR-365a-3p, and miR-409-3p in the control group	suppressor of bone resorption biomarker (CTX measurement)		
Seeliger *et al*. (2014)	RNA seq on pooled samples from 10 hip fracture patients with osteoporosis and 10 hip fracture patients without osteoporosis. For the validation 30 patients in each group were tested. Serum **non-based miRNAs profiling study in human**<br>PCR Array<br>SNORD96a and RNU6	83 different miRNAs, 51 were detectable in the serum of the osteoporotic patients with hip fractures<br>11 miRNAs ↑ in osteoporotic patients, 5 miRNAs ↑ identified in bone tissue osteoporotic patients 5 miRNAs increased in both serum and bone (miR-21, miR-23a, miR-24, miR-100 and miR-125b)	bioinformatic analysis of miRNA target and qPCR analysis in bone tissue samples of the patients miR-21, miR-23a, miR-24, miR-93, miR-100, and miR-148a, an osteogenic or osteoclastic involvement. downregulation of PDCD4 and an upregulation of c-FOS, both influenced by miRNA-21<br>The expression of RUNX2—possibly affected by miRNA-23a/miRNA-24-2/miRNA-27a complex	Independent cohort validation (60 serum samples and 40 bone tissue samples)<br>The associated area under the curve (AUC) was used to confirm the diagnostic value, highest value of 0.77 miR-122a (95% CI 0.69–0.86, p<0.0001), 0.63 for miR-21, miR-23a, miR-24, 0.68 for miR-93, 0.69 for miR-100, miR-124a, 0.76 for miR-125b, 0.61 for miR-148a.	Low

			miRNA-93 repressing the gene expression of osterix ↓<br>BMPR2 ↓under the influence of miRNA-100<br>VCAN ↓by miRNA-124a		
Sun *et al.*(2020)	6 osteoporotic patients with vertebral fractures, 6 osteoporotic patients without fractures and 6 healthy controls. Serum **non-biased miRNAs profiling study in human**<br>miRNA array	118 miRNAs ↑and 266 miRNAs↓ in osteoporotic samples versus controls<br>↓ miR-15b-5p, miR-92b-3p, miR-374c-3p, miR-144-5p, miR-148a-3p, miR-19b-3p, miR-106b-5p, miR-145-3p, miR-10b-5p in osteoporotic Individuals with stable fluorescent signals<br>miR-19b	Increase of miR-19b during osteoblastic differentiation<br>synthetic miR-19b promoted osteoblastic differentiation<br>PTEN (phosphatase and tenesin homolog deleted from chromosome 10) found to be directly repressed by miR-19b, with a concomitant increase in Runx2 expression and increased phosphorylation of AKT (protein kinase B, PKB).	Independent cohort validation (24 osteoporotic patients with fractures, 24 osteoporotic patients without fracture, and 24 healthy control)<br>miR-19b expression in mice bone tissues, human mesenchymal stem cells and osteoblasts<br>bone formation by miR-19b *in vivo*<br>ROC curves of miR-19b and osteoporosis without vertebral facture/ with fracture high sensitivity and specificity<br>miR-19b↑ during osteoblastic differentiation circulating miR-19b plays an important role in enhancing osteoblastogenesis,	Low
Weilner *et al.* (2015)	Serum from 19 postmenopausal women with osteoporotic	↑ miR-21 (not validated)	5 out of 7 tested miRNAs	Independent cohort validation (11 control and 12 fracture)<br>miR-22-3p, miR-328-3p, and let-7g-5p	Low

	fractures at the femoral neck or 18 control postmenopausal women **Non-biased miRNA profiling study** global mean of across all samples	↓ miR-10a-5p, miR-10b-5p, miR-133b, miR-22-3p, miR-328-3p, let-7g-5p at fracture	can modulate osteogenic differentiation of mesenchymal stem cells *in vitro*		
Yavropoulou *et al.* (2016)	Serum from postmenopausal women with osteoporosis/with fracture (n=35) or without fractures (n=35) **Targeted miRNA candidate study** quantitative PCR normalized with SNORD95, SORD96A, RNU6-2	1)↑ miR-124-3p, miR-2861 ↓miR-21-5p, miR-23a-3p, miR-29a-3p in OP vs non-OP 2) ↓ miR-21-5p in OP with VF Vs OP without VF	NA	No independent cohort validation miR-21-5p AUC 0.66 (95% CI 0.50, 0.81, P = 0.040), sensitivity 66%, specificity 71% miR-29a-3p AUC 0.61 (95% CI 0.45–0.77, P = 0.180) miR-23a-3p AUC 0.63 (95% CI 0.47–0.79, P = 0.115)	Low
Zarecki et al. (2020)	Serum from a total of 126 postmenopausal women 1) healthy controls (n = 42) 2) patients with low BMD and no fractures (n= 39) 3) patients with vertebral fractures and low BMD without a treatment against osteoporosis (n= 26)	↑ miR-375, miR-532-3p, miR-19b-3p, miR-152-3p, miR-23a-3p, miR-335-5p, miR-21-5p in P-MP women with VF/low BMD against without VF/low BMD and healthy controls miR-486-3p, miR-30e-5p, miR-127-3p, miR-214-3p, miR-550a-3p, miR-106b-	Measurement of bone turnover markers PINP correlated with miR-451a and miR-188-5p OC correlated with miR-19b-3p, BAP correlated with miR-532-3p, miR-19b-3p, CTX correlated with miR-19b-3p, miR-19b-3p; oestrogen	No independent cohort validation miR-375, miR-532-3p, miR-19b-3p, miR-152-3p, miR-23a-3p, miR-335-5p, miR-21-5p explain the difference between subjects with and without vertebral fractures by 72.7%	Low

4) patients with vertebral fractures and low BMD receiving a treatment for osteoporosis (n = 19)<br>**Targeted miRNA candidate study** quantitative PCR normalizing with UniSp4	5p, miR-133b, miR-143-3p at least one significant (P < 0.05) post-hoc test between any of the four group	signalling pathway (ESR1, ADCY1, ATF2, CALM1, PIK3R3, GNAQ PIK3CA)<br>miR-21-5p; Hippo signalling pathway (YAP1, SMAD7, LATS1, BMPR2)
